# 7aaRGD - a novel SPP1/integrin signaling-blocking peptide reverses immunosuppression and improves anti-PD-1 immunotherapy outcomes in experimental gliomas

**DOI:** 10.1186/s13046-025-03393-9

**Published:** 2025-04-25

**Authors:** Aleksandra Ellert-Miklaszewska, Paulina Pilanc, Katarzyna Poleszak, Adria-Jaume Roura, Salwador Cyranowski, Mitrajit Ghosh, Szymon Baluszek, Maria Pasierbinska, Bartłomiej Gielniewski, Julian Swatler, Yuliana Hovorova, Kamil Wojnicki, Bozena Kaminska

**Affiliations:** 1 Laboratory of Molecular Neurobiology, Nencki Institute of Experimental Biology, Warsaw, Poland; 2 Laboratory of Cytometry, Nencki Institute of Experimental Biology, Warsaw, Poland

**Keywords:** Glioma, Tumor microenvironment, Integrins, Immunotherapy, Blocking peptide, Glioma-associated macrophages

## Abstract

**Background:**

Immune checkpoint inhibitors (ICIs) present clinical benefits in many cancer patients but invariably fail in glioblastoma (GBM), the most common and deadly primary brain tumor. The lack of ICIs efficacy in GBM is attributed to the accumulation of tumor-reprogrammed glioma-associated myeloid cells (GAMs) that create a “cold” immunosuppressive tumor microenvironment (TME), impeding the infiltration and activation of effector T cells. GBM-derived αvβ3/αvβ5-integrin ligands, including SPP1, were shown to mediate the emergence of GAMs. We hypothesized that a combination strategy aiming to block the reprogramming of GAMs using a synthetic 7aaRGD peptide that targets SPP1/integrin signaling might overcome resistance to ICIs and reinvigorate anti-tumor immunity.

**Methods:**

Matrigel invasion assay was used to test the efficacy of 7aaRGD in glioma-microglia co-cultures. We determined the impact of 7aaRGD, administered as a monotherapy or combined with PD-1 blockade, on tumor growth, GAMs accumulation and phenotypes, arginase-1 levels and neovasculature in experimental gliomas. The effects of treatments on the tumor immune landscape were dissected using multiparameter flow cytometry, immunocytochemistry, cytokine profiling and RNA-seq analysis of sorted GAMs followed by CITE-seq based data deconvolution.

**Results:**

7aaRGD efficiently blocked microglia-dependent invasion of human and mouse glioma cells in vitro. Intratumorally delivered 7aaRGD alone did not reduce tumor growth in orthotopic gliomas but prevented the emergence of immunosuppressive GAMs and led to normalization of peritumoral blood vessels. Combining 7aaRGD with anti-PD-1 antibody resulted in reduced tumor growth, with an increase in the number of proliferating, interferon-ɣ producing CD8^+^T cells and depletion of regulatory T cells. Transcriptomic profiles of myeloid cells were altered by the combined treatment, reflecting the restored “hot” inflammatory TME and boosted immunotherapy responses. Intratumoral administration of 7aaRGD similarly modified the phenotypes of GAMs in human U87-MG gliomas in immunocompromised mice. Exploration of transcriptomic datasets revealed that high expression of integrin receptor coding genes in pre-treatment biopsies was associated with a poorer response to immune check-point blockade in patients with several types of cancers.

**Conclusions:**

We demonstrate that combining the blockade of SPP1/integrin signaling with ICIs modifies innate immunity and reinvigorates adaptive antitumor responses, which paves the way to improve immunotherapy outcomes in GBM.

**Graphical abstract:**

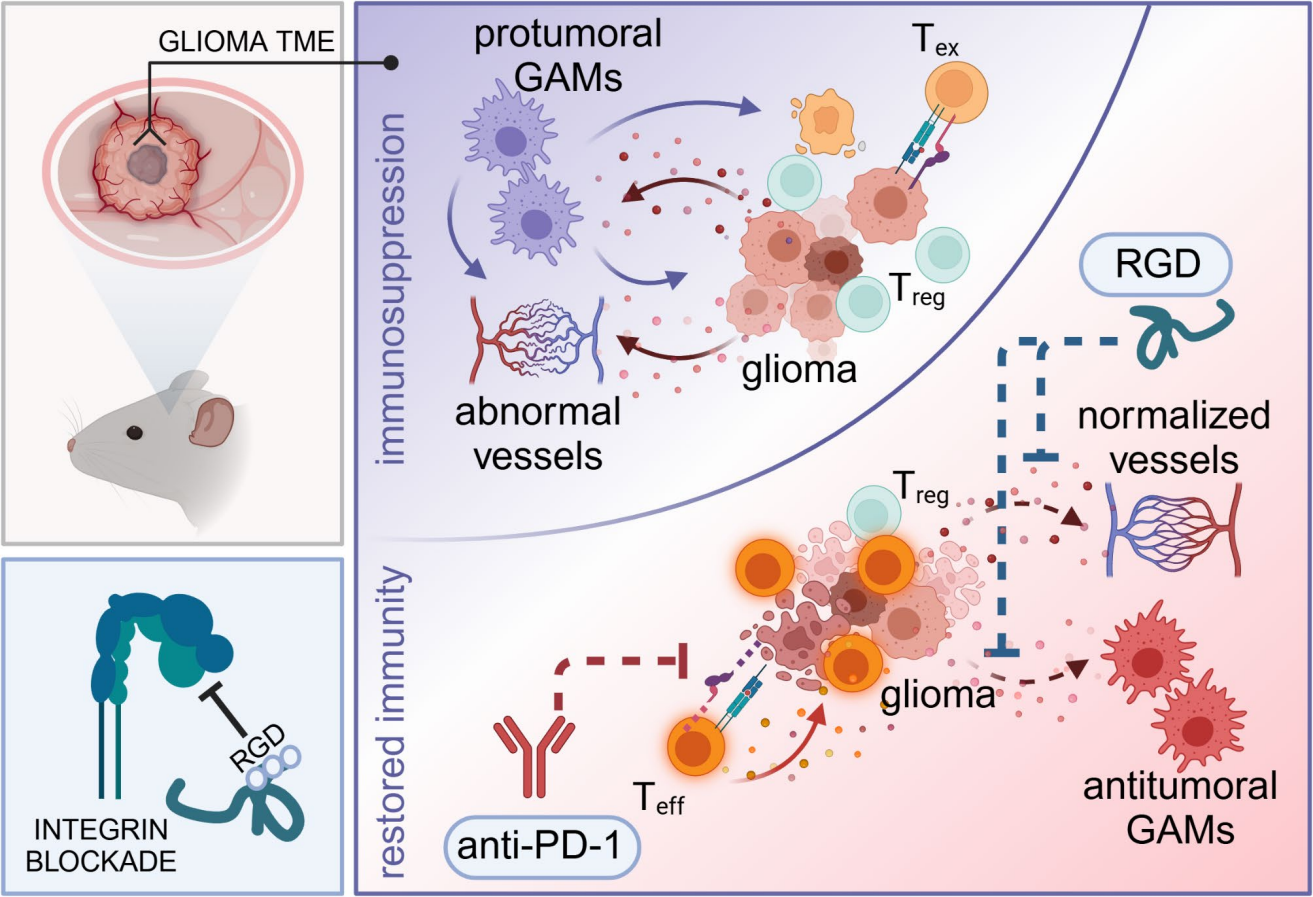

**Supplementary Information:**

The online version contains supplementary material available at 10.1186/s13046-025-03393-9.

## Background

Immune checkpoint inhibitors (ICIs) have altered therapeutic outcomes in a wide range of cancers, providing unparalleled survival benefits in some patients. Blocking antibodies against programmed cell death 1 (PD-1, CD279) or programmed cell death-ligand 1 (PD-L1, CD274) (anti-PD-1 or anti-PD-L1, respectively) are employed in more than 20 different indications [1]. Unfortunately, only approximately 20% of cancer patients (mostly those with melanoma and lung cancer) achieve long-term, durable responses with ICIs, while resistance is common in most patients [2]. Glioblastoma (GBM) is the most frequent and lethal primary brain tumor among adult-type diffuse gliomas [3]. Despite some promising results from phase II/III trials, ICIs have failed in phase III randomized trials in both newly diagnosed and recurrent GBM patients [4, 5]. This limited efficacy of ICIs is attributed to the highly immunosuppressive tumor microenvironment (TME) of GBM [6, 7].

GBM is known as a “cold” tumor with a low mutation burden, poor infiltration of effector T and NK cells, and an abundance of suppressive myeloid cells that may constitute of up to 30% of the tumor mass [7]. Human GBM and murine gliomas attract brain-resident microglia and bone marrow-derived myeloid cells, jointly called glioma-associated microglia and macrophages (GAMs), and impose tumor-supportive programs in these cells. In turn, GAMs increase tumor cell invasion and angiogenesis, and contribute to local immunosuppression by inhibiting the cytotoxic activity of T cells and initiating signals for their anergy or exhaustion, while promoting the expansion of protumorigenic T regulatory cells (Treg) [8–11]. Defective neoangiogenic blood vessels drive metabolic reprogramming in hypoxic niches and impair trafficking of T cells to tumors, which aggravates functional deficits in antitumor immunity and eventually contributes to ICI therapy failure [12–14]. Expermental studies have shown that combination strategies aiming to reverse the protumor reprogramming of myeloid cells and to normalize the tumor neovasculature may overcome TME-instigated resistance to immunotherapy [15–19].

Multiple approaches, including targeting GAMs with an inhibitor of the colony-stimulating factor (CSF)-1 receptor [20] or CCR2/CCL2 axis blockade [16], have been proposed to modify the glioblastoma TME to improve therapeutic responses. We previously identified two glioma-derived αvβ3/αvβ5-integrin receptors ligands, osteopontin/secreted phosphoprotein 1 (SPP1) and lactadherin/milk fat globule-epidermal growth factor 8 (MFG-E8), as activators of myeloid cell reprogramming in gliomas [21]. Silencing of *Spp1* or *Mfge8* expression in glioma cells prevented GAMs accumulation and reduced tumor growth in orthotopic rat gliomas [21]. Congruently, SPP1 was shown to recruit GAMs to mouse gliomas via αvβ5 integrin, and systemic administration of osteopontin-eliminating aptamers increased the median survival time of tumor-bearing animals by 68% [22]. Interestingly, SPP1 and MFG-E8 share their RGD (arginine–glycine–aspartate) αvβ3/αvβ5-binding motif with another GBM-secreted integrin ligand, periostin (POSTN). POSTN drives the recruitment and maintains the immunosuppressive phenotype of GAMs, and its genetic depletion had anti-glioma effects [23, 24]. Despite the well-described role of integrins in cancer, their expoitation in theranostics and several clinical attempts to use integrin inhibitors as anti-angiogenic agents [25–27], the effects of integrin blockade on the functional regulation of tumor-infiltrating myeloid cells have been underexplored. As a strategy to prevent the reprogramming of myeloid cells into GAMs, we designed a synthetic 7-amino-acid peptide with the RGD motif (‘7aaRGD’), which competitively blocked the interactions of SPP1 and MFG-E8 with the integrins, abolished the activation of the focal adhesion kinase-dependent signaling pathway, decreased microglial phagocytosis and migration, and reduced the expression of proinvasive phenotype genes in glioma secretome-stimulated microglia [21].

In this study, we demonstrate that 7aaRGD reduces microglia-dependent invasion of murine and human glioma cells in vitro and has a bimodal antitumor action in vivo. Upon intratumoral administration to orthotopic gliomas, 7aaRGD shifts the glioma TME toward a “hot” phenotype by blocking the reprogramming of GAMs, increasing the percentage of CD8 + T cells among infiltrating lymphocytes, and normalizing the tumor vasculature. As a monotherapy, 7aaRGD does not reduce tumor growth, however, co-administration of the peptide with an anti-PD-1 antibody ultimately augments proinflammatory responses of myeloid cells and unleashes cytotoxic T cells activity, which leads to eradication of the tumors. We provide evidence that combining integrin blockade with ICIs reinvigorates antitumor immunity by boosting both innate and adaptive immune responses, thus indicating a novel strategy to increase the efficacy of immunotherapy. These results provide a new perspective for the treatment of GBM patients and pave the way for improving the responses to ICIs in GBM and other cancers.

## Methods

### Cell cultures

Murine glioma GL261 cells were obtained from Prof. Helmut Kettenman (MDC, Berlin, Germany) and modified to GL261 tdTomato + luc + glioma cells as described previously [11]. The murine and human glioma cell lines: U251-MG, U87-MG (ATCC, Manassas, VA) and U87-MG RFP+ (AntiCancer Inc., San Diego, CA, USA) were cultured in Dulbecco’s modified Eagle’s medium (DMEM) supplemented with 10% fetal bovine serum (FBS) (Gibco, MD, USA). After thawing, the GL261 tdTomato + luc + and U87-MG RFP + cells were supplemented with 400 µg/ml G418 (Roche, Manheim, Germany) for two passages. Stable transfection of U87-MG RFP + cells with pSilencer 2.1-U6hygro plasmid encoding a non-targeting shRNA or a specific SPP1-targeting hairpin shRNA generated shNeg and shSPP1 cells, respectively. Human SV40 immortalized microglia (HMSV40) were cultured in PriCoat T25 flasks in Prigrow III medium (Applied Biological Materials, Richmond, Canada) supplemented with 10% FBS (Gibco, MD, USA). Murine immortalized microglial BV2 cells (received from Prof. Klaus Reymann from the Leibniz Institute for Neurobiology) were cultured in DMEM GlutaMAX™ supplemented with 2% FBS (Gibco, MD, USA). Primary mouse microglia cultures were prepared as described [28]. All the cells were cultured with antibiotics (100 U/ml penicillin and 100 µg/ml streptomycin) in a humidified atmosphere of CO_2_/air (5%/95%) at 37 °C (Heraeus, Hanau, Germany). Mycoplasma contamination-free status was checked regularly.

### Cell viability and proliferation assays

Cell viability was evaluated using the MTT metabolism test. Cell proliferation was measured using a BrdU ELISA kit (Roche, Mannheim, Germany) according to the manufacturer’s protocol. Briefly, 5 × 10^3^ cells were seeded onto 96-well plates. The next day, the media was changed to DMEM GlutaMAX™ supplemented with 2% FBS (Gibco, MD, USA), and the 7aaRGD and 7aaRAE peptides were added at a final concentration of 100 µM for 18 h. Peptides with N-terminal acetylation and C-terminal amidation were purchased from GenScript (Rijswijk, Netherlands). The peptides were dissolved in DMSO, and 0.2% solvent was added as a control. MTT solution (0.5 mg/mL; Sigma‒Aldrich, Taufkirchen, Germany) or BrdU was added for 1.5 and 2 h, respectively. Optical densities were measured at 570 nm for the MTT assay and at 450 nm for the BrdU assay using a scanning multiwell spectrophotometer.

### Invasion assays

The invasion assay was performed as previously described [28]. BV2 and HMSV40 cells (4 × 10^4^) were plated onto a 24-well plate and co-cultured with GL261 cells (1 × 10^5^/insert), U87-MG and U251 cells (4 × 10^4^/insert) on Matrigel-covered inserts in 2% FBS-containing media. The cells were treated with 100 µM 7aaRGD and 7aaRAE (control) peptides or 0.2% DMSO. GL261 cells were co-cultured with BV2 cells for 24 h, and human glioma cells were cultured with BV2 or HMSV40 cells for 18 h. Invading cells fixed on the membranes were stained with DAPI.

### Peptide stability

The 7aaRGD peptide was dissolved in water at a concentration of 2 mg/ml and incubated in osmotic pumps for 1, 7 and 14 days at 37 °C. The peptide concentration was measured using Jupiter Proteo C12 2.1 × 250 mm, 4 μm column (Phenomenex, Torrance, USA) and HPLC Prominence coupled LCMS-IT-TOF (Shimadzu, Duisburg, Germany) under the following conditions: phase A: 0.1% HCOOH in Milli-Q water; phase B: 0.1% HCOOH in ACN; and gradient conditions: 0 min 2% B, 20 min 50% B, 25 min 95% B, 30 min 95% B, 35 min 5% B, and 55 min 5% B at a 0.2 ml/min flow rate. One milliliter of sample was injected. A Shimadzu IT-TOF ESI-MS system was used for mass analysis in automatic mode with a scan range of 150–2000 Da and an ion accumulation time of 10 ms. Electrospray ionization was performed in positive ionization mode with a spray capillary voltage of 5.0 kV. The interface temperature was maintained at 250 °C, and the heat block temperature was 520 °C. Nebulizing gas was introduced at 1.5 L/min, and the drying gas pressure was set to 100 kPa. Mass spectra were recorded in positive ion mode using the LCMSsolution software provided by Shimadzu. The analysis was performed at the Department of Chemistry of Warsaw University, Poland.

### Stereotactic implantation of glioma cells and treatments

Male C57BL/6 mice or Athymic Nude-Foxn1nu mice (Charles River Laboratories, USA) (10–12 weeks old) were housed with free access to food and water on a 12 h/12 h day/night cycle. All efforts were made to minimize the number of animals and animal suffering. The mice were anesthetized with isoflurane (4–5% induction, 1–2% maintenance) via an isoflurane vaporizer (Temsega, Tabletop Anesthesia Station). Before starting the surgical procedure and during the surgery, the depth of anesthesia was verified. The choice of anesthetics was recommended by a veterinarian and approved by the local Ethics Committee. GL261 tdTomato^+^luc^+^ glioma cells (80,000 in 1 µL of DMEM) in C57BL/6J or U87-MG RFP (50,000 in 1 µL of DMEM) in Athymic Nude-Fox1nu mice were stereotactically injected into the right striatum at the following coordinates: 1 mm anterior and 2 mm lateral from bregma, 3 mm deep from the surface of the brain. At the same time, Alzet osmotic micropumps (DURECT Corporation, Cupertino, CA, USA) were installed in a subcutaneous pocket on the back, slightly caudal to the scapulae. A small incision was made in the shaved skin, and a hemostat was used to create a subcutaneous pocket for the pump, which was then inserted into the pocket, and the wound was closed with tissue glue. Prior to implantation, the metal flow moderators were replaced with PEEK flow moderators to be MRI compatible. To ensure that the pumps were active when implanted, the filled pumps were placed in sterile saline at 37 °C for 24 h before implantation. Osmotic pumps were filled with H_2_O (vehicle), 7aaRGD or 7aaRAE peptides at a concentration of 2 mg/ml in H_2_O, and by means of a catheter, they continuously delivered the solutions intratumorally at a controlled rate of 0.11 µL/h for 21 or 28 days. All experiments in C57BL6 mice were conducted after implantation of GL261tdTomato + luc + cells, except for the experiment evaluating FITC-labelled peptide distribution, in which we used GL261 cells.

The mice were monitored until they completely recovered from anesthesia. The animals were weighed weekly and observed daily for clinical symptoms and evidence of toxicity by evaluating their eating, mobility, weight loss, hair loss, and hunched posture. Anti-PD-1 antibody (BioLegend, GoInVivo™ Purified anti-mouse CD279) was injected intraperitoneally (i.p.) at a dose of 10 mg/kg on days 8, 10, 12, and 14 post-implantation. The control groups received IgG antibody by i.p. injection. Animals were euthanized when they lost more than 20% of their body weight at day 0.

### Tumor size measurement using magnetic resonance imaging

The heads of the animals were scanned with a 7T BioSpec 70/30 MR system (Bruker, Ettlingen, Germany) equipped with Avance III console and an actively shielded gradient system B-GA 20 S (amplitude 200 mT/m) with an integrated shim set up to the 2nd order. A combination of a transmit cylindrical radiofrequency volume coil (8.6 cm inner diameter, Bruker) and a head-mounted mouse dedicated receive-only array coil (2 × 2 elements, Bruker) was used. The animals were anesthetized with 1.5–2% isoflurane (Baxter, Deerfield, IL, USA) in oxygen and positioned prone with the head placed in the MR-compatible bed integrated with an anesthesia mask. Respiration and rectal temperature were monitored throughout the experiment with an MR-compatible small animal monitoring system (SA Instruments, Stony Brook, NY, USA). All the imaging sessions started with a localizer protocol consisting of three orthogonal scout scans to accurately position the animal inside the magnet center. To evaluate the volumes of the brain structures, structural transverse MR images covering the whole brain were acquired with T2-weighted TurboRARE (TR/TEeff = 7000/30 ms, RARE factor = 4, spatial resolution = 86 μm × 86 μm × 350 μm, 42 slices, no gaps, number of averages (NA) = 4, scan time ~ 23 min). MRI scans were evaluated using OsiriX software (Pixmeo, Geneve, Switzerland), and manually delineated tumor regions in the image series were used for volumetric assessments.

### Cytokine analysis

Pro- and anti-inflammatory cytokines were measured in serum and brain homogenates from control and treated animals. Blood was collected before perfusion and allowed to clot for 30 min before centrifugation (1000 × g, 10 min at room temperature). The serum was collected and stored at -80 °C. Brain homogenates were prepared by adding an equal volume of Cell Lysis Buffer 2 (R&D Systems, Minneapolis, MN, USA) to dissociated brain tissues. The samples were then incubated at room temperature for 30 min with gentle agitation, and debris were removed via centrifugation. The levels of cytokines were measured using the Luminex Assay Mouse Premixed Multi-Analyte Kit (R&D Systems, Minneapolis, MN, USA) according to the manufacturer’s protocol. Cytokine levels were determined using the MAGPIX Multiplexing Instrument (Luminex, TX, USA) with XPonent software. The results for each cytokine are expressed as pg/ml for serum samples and pg/mg protein for brain homogenates. The protein concentration in the brain homogenates was estimated using Bradford reagent (Sigma Aldrich), and the optical density at 570 nm was measured using a scanning multiwell spectrophotometer.

### Immunohistochemistry on brain slices

The animals were sacrificed 21 days after GL261 tdTomato^+^luc^+^ cell implantation and perfused with 4% paraformaldehyde in phosphate-buffered saline (PBS). The brains were removed, fixed for 48 h in the same fixative solution and placed in 30% sucrose in PBS at 4 °C until the tissue sank to the bottom of the flask. The tissue was frozen in Tissue Freezing Medium (Leica Biosystems, Richmond, IL, USA) and cut into 12 μm coronal sections using a cryostat. The slides were dried at room temperature for 2 h after being transferred from − 80 °C storage. The cryosections were blocked in PBS containing 10% donkey serum and 0.1% Triton X-100 for 2 h and incubated overnight at 4 °C with rabbit anti-IBA1 and goat anti-Arg1 antibodies or FITC-conjugated *Lycopersicon esculentum* (Tomato) lectin (Vector Labs, FL-1171-1). Next, the sections were washed in PBS and incubated with the corresponding secondary antibodies for 2 h at room temperature. All the antibodies were diluted in 0.1% Triton X-100/PBS solution containing 3% donkey serum. Nuclei were counterstained with DAPI (0.001 mg/ml). Images were acquired using an Olympus microscope (Fluoview, FV10i) and a Leica DM4000B fluorescence microscope. For reagent specifications, catalog numbers, and concentrations, see Table S1. We quantified the percentage of IBA1^+^ Arg1^+^ cells in 3 different areas of the tumor per animal. For lectin staining, three different sections from each mouse brain were analyzed. In each section, 2 regions of interest (ROIs) on the ipsilateral side (near the tumor core) were analyzed.

### Tissue dissociation, flow cytometry and FACS sorting

On day 21 or 28 after GL261-tdTomato^+^luc^+^ or U87-MG RFP + cell implantation, the mice were perfused transcardially with cold PBS prior to excision of the brain and spleen. The tumor-bearing hemispheres were dissociated enzymatically with collagenase type IV and DNase I (both from Merck, Darmstadt, Germany) at final concentrations of 2.5 mg/ml and 0.5 mg/ml, respectively, using gentleMACS Octo Dissociator (Miltenyi Biotec) according to the manufacturer’s protocol. Next, the enzymatic reaction was stopped by the addition of Hank’s Balanced Salt Solution with calcium and magnesium (Gibco, Germany). The resulting single-cell suspension was filtered through 70 μm and 40 μm strainers and centrifuged at 300 × g and 4 °C for 10 min. Myelin was removed by density gradient centrifugation in 22% Percoll as described previously [35]. Next, the cells were collected, washed with PBS and counted using NucleoCounter (Chemometec, Gydevang, Denmark).

The spleen was passed through a 70 μm strainer and gently ground using the plunger of a syringe with 3 ml of PBS to yield a single-cell suspension. Following centrifugation (300 × g, 5 min), red blood cells were lysed using ACK Lysing Buffer (Life Technologies, Grand Island, NY, USA) for 10 min at room temperature. The splenocytes were collected via centrifugation, washed with PBS and counted using NucleoCounter (Chemometec, Gydevang, Denmark).

For preparation for flow cytometry analysis, samples were handled on ice and protected from light exposure. Prior to staining with antibodies, the samples were incubated with eFluor 506 fixable viability dye (Thermo Fisher) in PBS for 10 min. Next, the samples were incubated for 10 min with rat anti-mouse CD16/CD32 Fc Block™ (BD Pharmingen) in Stain Buffer (BD Pharmingen) to block FcγRIII/II and reduce nonspecific antibody binding. Then, the cell suspensions were incubated for 30 min with an antibody cocktail in Stain Buffer (BD Pharmingen) for the detection of surface antigens. For intracellular staining, the cells were fixed and permeabilized (Foxp3 fixation/permeabilization buffer, eBioscience) prior to incubation with antibodies. For a list of antibodies, see Table S1. For FACS sorting, the cells were stained with an anti-CD11b (M1/70 clone) antibody labeled with FITC (BD Pharmingen) and an anti-CD45 (30-F1 clone) antibody labeled with PE-Cy7 (BD Pharmingen).

For intracellular cytokine staining, freshly isolated cells from the tumor-bearing brain hemispheres were resuspended in stimulating culture media supplemented with 50 ng/ml PMA, 1 µg/ml ionomycin (Sigma Aldrich) and protein transport inhibitor cocktail (brefeldin A and monensin at final concentrations of 10.6 µM and 0.2 mM, respectively; Life Technologies, Carlsbad, CA, USA) for 4 h and then processed for staining of surface and intracellular antigens as described above.

All the antibodies were titrated prior to staining to establish the amount yielding the best stain index. Data were acquired using a BD LSR Fortessa Analyzer cytometer and analyzed with FlowJo software (v. 10.5.3, FlowJo LLC, BD). Gates were set on the basis of FMO (fluorescence minus one) controls and back-gating analysis. The percentages on the cytograms are given as the percentage of a parental gate. For computational analyses, each sample was downsampled to obtain 10,000 CD45^+^CD11b^+^ or CD45^+^CD11b^−^ cells. For tSNE, all samples were concatenated and processed using specific plugins in FlowJo v10.

CD11b^+^ cells were FACS-sorted from naïve or tumor-bearing hemispheres (pooled from 2 animals per sample at day 21 and from individual mice at day 28) using Cell Sorter BD FACSAria II. All flow cytometry experiments were performed at the Laboratory of Cytometry, Nencki Institute of Experimental Biology. For reagent specifications, catalog numbers and dilutions, see Table S1. The gating strategies used in the analysis are shown in Fig. S10 and Fig. S11.

### RNA isolation, mRNA library preparation and RNA sequencing

Immediately after sorting, CD11b^+^ cells were centrifuged and lysed for further isolation of RNA using the RNeasy Plus Mini Kit (Qiagen, Germany) according to the manufacturer’s protocol. The integrity and quality of the RNA were assessed on an Agilent 2100 Bioanalyzer with an RNA 6000 Pico Kit (Agilent Technologies, CA, USA). Strand-specific RNA libraries were prepared for sequencing (3–4 biological replicates/treatment) using a KAPA Stranded mRNA-Seq Kit (Kapa Biosystems, MA, USA). Poly-A mRNAs were purified from 100 ng of total RNA using poly-T-oligo-magnetic beads (Kapa Biosystems, MA, USA). mRNAs were fragmented, and first-strand cDNA was synthesized using reverse transcriptase and random hexamers. Second-strand cDNA synthesis was performed by removing RNA templates and synthesizing replacement strands, incorporating dUTP in place of dTTP to generate double-stranded (ds) cDNA. The dsDNA was then subjected to the addition of “A” bases to the 3′ ends and the ligation of adapters from NEB, followed by uracil digestion by USER enzyme (NEB, MA, USA). The amplification of fragments with adapters ligated at both ends was performed by PCR using primers containing TruSeq barcodes (NEB, Ipswich, MA, USA). The final libraries were analyzed using a Bioanalyzer and Agilent DNA High Sensitivity chips (Agilent Technologies, Santa Clara, CA, USA) to confirm the fragment sizes (~ 300 bp). Quantification was performed using a Quantus fluorometer and the QuantiFluor dsDNA System (Promega, Madison, Wisconsin, US). Libraries were loaded onto a rapid run flow cell at a concentration of 8.5 pM onto a rapid run flow cell and sequenced on an Illumina HiSeq 1500 paired-end platform.

### Data processing and analysis

Illumina-specific adapters, short reads, and low-quality 5′ and 3′ bases were filtered out in the FASTQ files using the Trimmomatic [10.1093/bioinformatics/btu170] tool (version 0.36). The resulting RNA sequencing reads were aligned to a reference mouse genome sequence (mm10) with STAR aligner [29] (version 2.6.1b) using the two-pass Mode Basic option. Duplicate reads were then identified and flagged using Picard Tools (version 2.17.1) [broadinstitute.github.io/picard/]. The quantification of mapped reads and summarization by gene was performed using HTSeq-count (version 0.11.1), with paired mode (-p) and reverse stranded mode (-s reverse) enabled, and only reads with MapQ values of 10 or higher were considered. Low-expressed features were filtered out, and an analysis of differentially expressed genes was performed using NOIseq. Only mRNAs encoding protein-coding genes were retained for downstream analysis.

To identify transcriptomic differences between groups, differential expression analysis was performed using NOIseq methods, with the control (Vehicle) as the reference group compared with 7aaRGD, 7aaRAE or 7aaRGD, anti-PD-1 and combination of 7aaRGD + anti-PD-1. The variance stabilizing transformation (vst function) was used for visualization. Pathway enrichment analysis was performed using fgsea [30] on genes ranked by the fold change. Gene Ontology Biological Processes (GO: BP) was used to better understand the mechanistic findings of the enriched gene lists. The clusterProfiler, VennDiagram and ggplot2 R packages were used to visualize the data.

Bulk RNA-seq data deconvolution was performed using the bisque deconvolution tool [31] with default settings. Deconvolution was based on the cell type-specific transcriptomic signatures generated in our laboratory using CITE-seq on isolated brain CD11b^+^ cells derived from the same glioma model in mice [32].

### Statistical analysis

All in vitro data represent at least three independent experiments performed in duplicate or triplicate. The statistical significance of the invasion assay results was calculated using the chi-square test. The numbers of animals per group are specified in the figure captions. Comparisons between two groups were performed with a two-tailed Student’s t test. For multigroup comparisons, one-way analysis of variance (ANOVA) was used, followed by Tukey’s HSD test. All the statistical analyses were performed using GraphPad Prism 7.0 software. *P* < 0.05 was considered statistically significant.

## Results

### 7aaRGD peptide blocks microglia-induced invasion of murine and human glioma cells

**Fig. 1:**
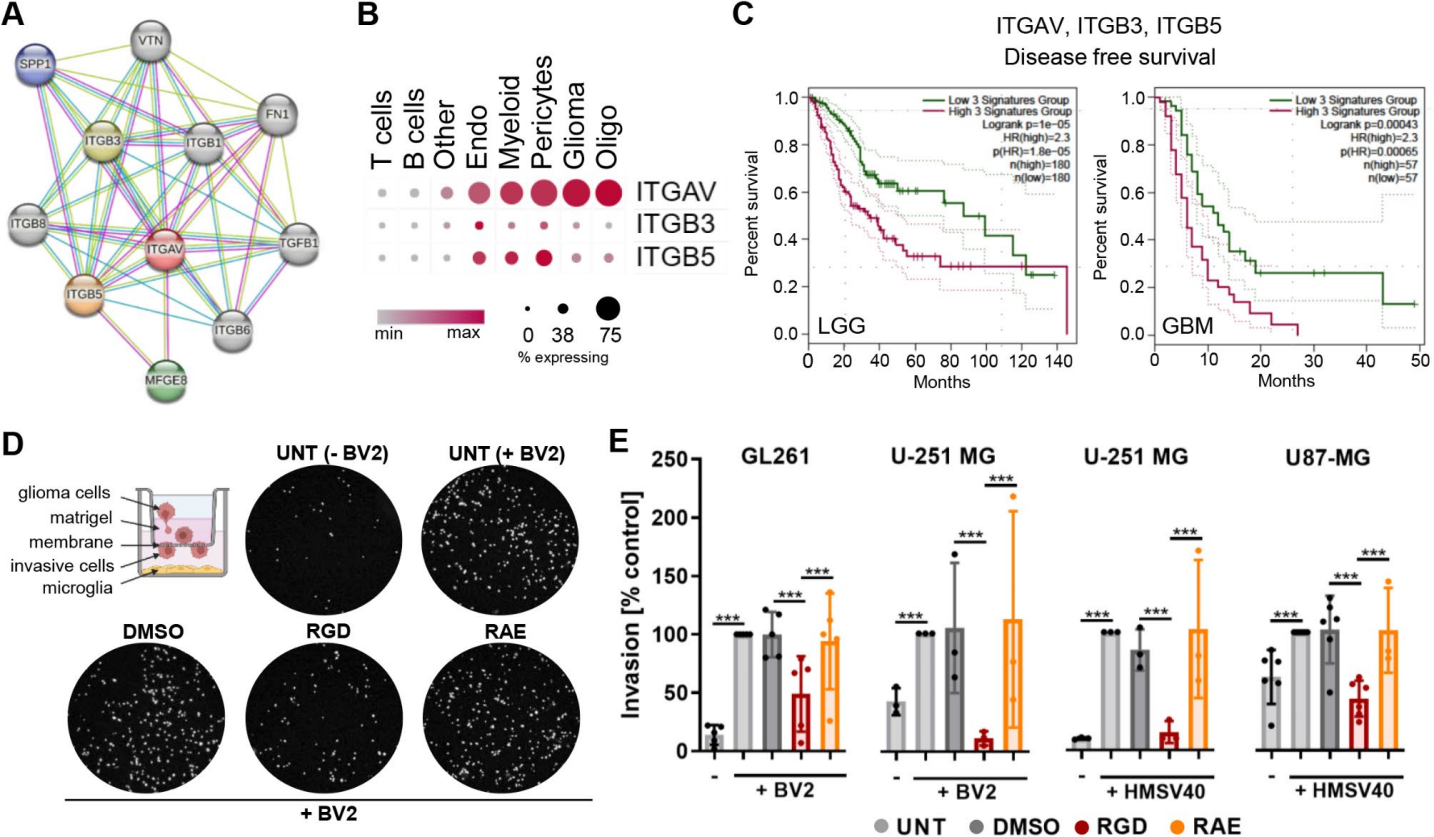
The 7aaRGD peptide abrogates microglia-dependent glioma cell invasion. (**A**) The top 10 predicted associations of integrin αv (ITGAV)-interacting proteins modeled using STRING. The purple line represents experimental evidence, the yellow line– text mining evidence, and the light blue line - database evidence. (**B**) Scaled mean expression of integrin-coding genes in the indicated cell types in scRNA-seq of 44 human gliomas (GSE182109 study, SingleCell portal). (**C**) Kaplan‒Meier curves generated using GEPIA2 for low-grade glioma (LGG)- and GBM patients with high (in purple) and low (in green) expression levels of a multigene signature (cutoff: high, 65%; low, 35%; the dotted line depicts the 95% CI). (**D-E**) Matrigel invasion assay. Glioma cell invasion in the absence or presence of microglia (see the scheme) was measured after 18 h of co-culture. (**D**) Representative images of the DAPI-stained nuclei of invading GL261 cells in co-culture with BV2 microglia; the cells were untreated (UNT) and treated with solvent (DMSO, 0.2%) or 100 µM 7aaRGD or 7aaRAE peptides. (**E**) Quantification of invasion of GL261, U-251 MG and U87-MG glioma cells co-cultured with murine BV2 or human HMSV40 microglia in the presence of DMSO, 7aaRGD or 7aaRAE peptides. The invasion of untreated glioma cells (UNT) co-cultured with microglia was set as 100%. All the experiments were performed three times in duplicates. Statistical significance was calculated using the chi-square test; ***p value 0.001

The integrin-blocking peptide (7aaRGD) was designed to block the binding of glioma-derived SPP1 and MFGE8 to αvβ3/αvβ5 integrins [21]. Interactome analysis of the human integrin αv generated by STRING (Fig. 1A) revealed SPP1 and MFG-E8 among the top 10 proteins with experimentally confirmed physical associations with these integrins via complexes formed with the β3 and β5 subunits. By analyzing publicly accessible scRNA-seq data from human gliomas [33] via the SingleCell portal, we detected the predominant expression of *ITGAV*, *ITGB3* and *ITGB5* (encoding αv, β3 and β5 integrins, respectively) in clusters of myeloid cells, pericytes and endothelial cells (Fig. 1B). These findings indicate that glioma-associated myeloid cells and blood vessels might be the main targets of integrin blockade with the 7aaRGD peptide. Moreover, we explored TCGA expression datasets of low- and high-grade gliomas. The levels of *ITGAV*, *ITGB3* and *ITGB5* were significantly higher in GBM samples than in non-tumor specimens (Fig. S1A). *ITGAV* and *ITGB5* expression was also elevated in astrocytomas. Among all GBM subtypes, the expression of these genes was the highest in the mesenchymal GBMs, which have been associated with the worst clinical outcome (Fig. S1B). Survival analysis based on the expression of individual integrin genes (Fig. S1C) or a multigene signature (Fig. 1C) revealed that high levels of *ITGAV*, *ITGB3* and *ITGB5* correlate with a poor prognosis in glioma patients. These data provided a rationale for further exploration of the efficacy of integrin-blocking peptides in the treatment of GBM.

We previously assessed the impact of the 7aaRGD integrin-blocking peptide on phenotypic profile and functionalities of rat microglia cultures [21]. To further validate the findings in vitro, we investigated whether 7aaRGD prevents the tumor cells-induced activation of microglia, and primarily its ability to stimulate glioma cells invasion. The co-cultures of microglia and glioma cells mimic their interactions within the tumor microenvironment. Glioma cells reprogram microglia, which in turn support glioma growth and invasion and several microglia-driven mechanisms have been implicated in these processes in vitro, in organotypic brain slices and in vivo [21, 34–36]. *Tgfbi* and *Cxcl14*, are two of the top upregulated genes in primary mouse microglia exposed to U87-MG-conditioned media that are related to cell chemotaxis and extracellular matrix organisation [28]. As 7aaRGD is expected to block SPP1/integrin signaling, we tested its efficacy in co-cultures of primary mouse microglia with U87-MG glioma cells, which were stably transfected with either a control shRNA (shNeg) or an SPP1-targeting shRNA to achieve *SPP1* gene silencing (shSPP1). 7aaRGD reduced the upregulated expression of *Tgfbi* and *Cxcl14* genes in microglia only in the presence of shNeg cells. The induction of these genes was lower in co-cultures with shSPP1 cells and was not affected by 7aaRGD (Fig. S2A). Additionally, 7aaRGD downregulated the expression of at least one of the known invasion-promoting factors in BV2 microglia cells co-cultured with GL261 or human U87-MG glioma cells (Fig. S2B). The differences in the responses stem from the use of two distinct co-cultures systems. Of note, in microglia co-cultured with either of the glioma cells, 7aaRGD treatment led to increased levels of *iNos*, one of the canonical M1 phenotype markers, corroborating our previous observation in rat microglia [21].

Next, we evaluated the effects of the designed 7aaRGD peptide and a control 7aaRAE peptide on the microglia-dependent invasion of murine (GL261) and human (U251-MG, U87-MG) glioma cells. In the 7aaRAE peptide, the RGD motif (arginine-glycine-aspartic acid) is changed to RAE (arginine-alanine-glutamic acid), which blunts its activity. Co-culture of glioma cells with mouse BV2 or human HMSV40 microglia enhanced the invasion of glioma cells through the Matrigel layer (Fig. 1D-E, S2C). The 7aaRGD peptide (and not the 7aaRAE) significantly reduced the microglia-induced invasion of glioma cells and was effective in all cell culture settings (including human glioma cells co-cultures with either murine or human microglia) (Fig. 1D-E).

The peptides exhibited no cytotoxic effects on glioma cells or microglia, as evidenced by the cell viability (MTT metabolism, Fig. S2D) and proliferation (BrdU incorporation, Fig. S2E) assays. These data confirm that the inhibitory effect of the 7aaRGD peptide on microglia-supported glioma invasion is not due to its toxicity but stems from peptide interference in glioma-microglia communication and reprogramming.

### 7aaRGD alleviates the emergence of the tumor-supportive TME in gliomas

Next, we sought to determine whether the 7aaRGD peptide affects the reprogramming of myeloid cells by gliomas in vivo and whether it interferes with tumor growth in mice. GL261 cells implanted into the striatum of immunocompetent C57BL/6J mice recapitulate many characteristics of human GBMs and are frequently used in preclinical glioma research [37]. The peptide was administered intratumorally via osmotic micropumps installed at the time of glioma cell implantation (Fig. S3A).

HPLC combined with mass spectrometry analysis of the 7aaRGD peptide incubated in the osmotic pumps at 37 °C revealed no products of degradation or any decline in the peptide level throughout the incubation period, indicating that the peptide was stable in water and did not bind to the pump itself (Fig. S3B). According to the biodistribution studies, an intratumorally administered FITC-labeled 7aaRGD peptide was present in the tumor core and diffused to the surrounding tissue, indicating efficient delivery and penetration within the brain parenchyma (Fig. S3C).

**Fig. 2:**
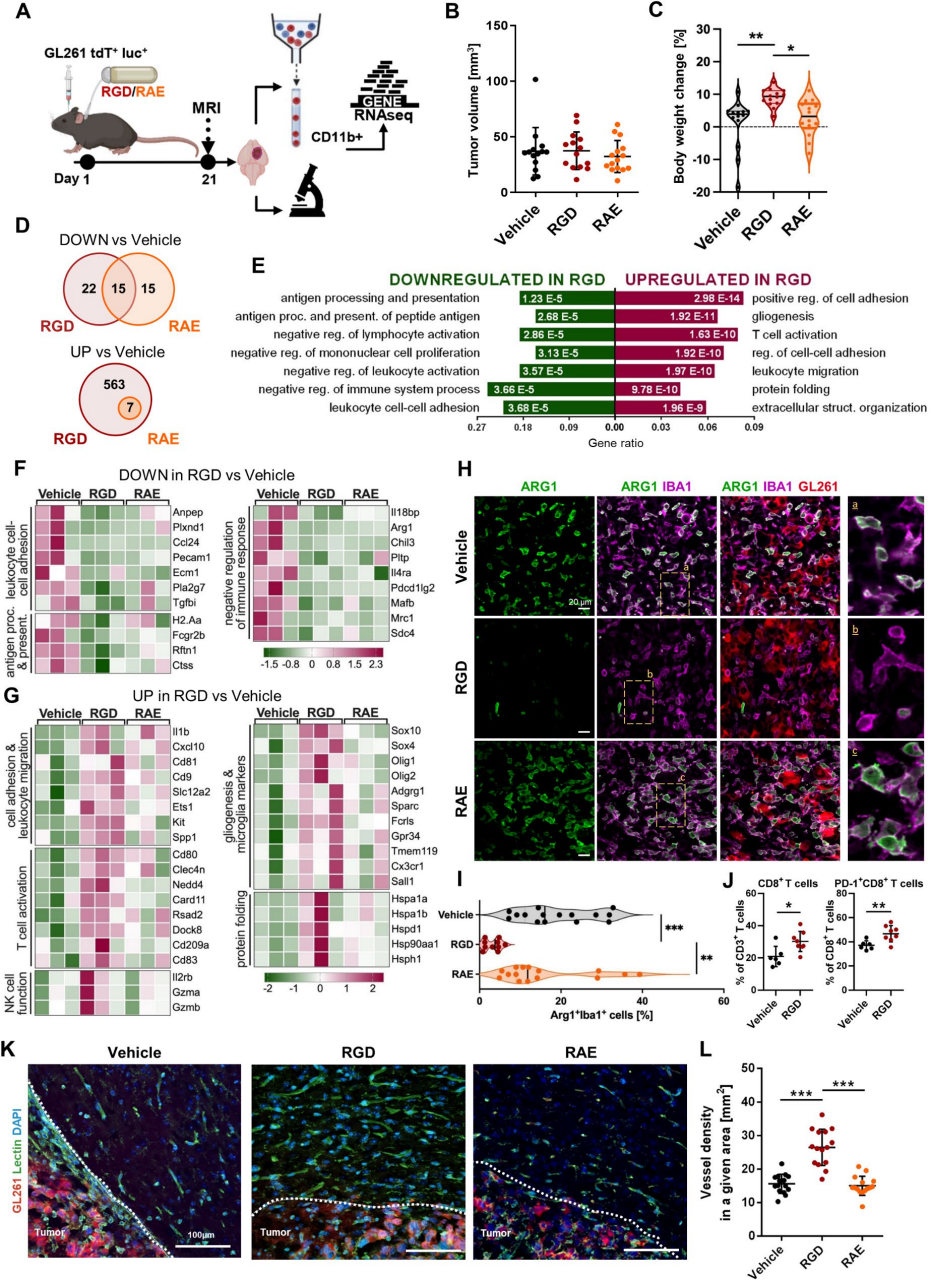
Intratumorally delivered 7aaRGD modifies the tumor microenvironment. (**A**) Schematic representation of the experimental pipeline. (**B**) Mice implanted with GL261 tdTomato + luc + glioma cells received water (Vehicle), 7aaRAE (RAE) or 7aaRGD (RGD) peptide via osmotic pumps. Tumor volume was quantified by MRI at 21 DPI; *n* = 15–16 per group. (**C**) Comparison of mouse body weights between 0 and 21 DPI; *n* = 15–16; the line is plotted as the mean. (**D-G**) RNA-seq gene expression profiling of CD11b^+^ cells from the tumor-bearing hemispheres of mice at 21 DPI. (**D**) Venn diagrams showing a number of downregulated and upregulated DEGs in CD11b^+^ cells from the 7aaRGD and 7aaRAE groups compared to the vehicle group. (**E**) Functional enrichment analysis with Gene Ontology (GO) biological processes for up- and downregulated genes in the 7aaRGD group compared to Vehicle. The enriched GO pathways (selected from the top ranked) are shown, and the size of the bars indicates the gene ratios (the number of genes annotated to the pathway/total number of DEGs with adjusted *p* values < 0.05). Z-score heatmaps for selected down- (**F**) and upregulated (**G**) genes in CD11b^+^ cells from 7aaRGD- vs. Vehicle-treated animals. (**H**) Representative images of glioma-bearing brains (at 21 DPI) stained with anti-IBA1 (in purple) and anti-Arg1 (in green) antibodies and co-stained with DAPI are shown. (**I**) Quantification of IBA1^+^Arg1^+^ cells. The average values from 3 fields are presented; *n* = 5. (**J**) Percentages of CD8^+^ T cells and PD-1^+^CD8^+^ T cells isolated from tumor-bearing brains in all experimental groups. (**K**) Representative images of sections from glioma-bearing brains stained with lectin (green) and co-stained with DAPI are shown. (**L**) Quantification of vessel density. Three independent sections from each mouse brain were analyzed; in total 15 sections from each group. Each dot represents an average density in 2 regions of interest (ROIs, see Fig. S4D), both near the tumor core. Significance was calculated with One-Way ANOVA, followed by Tukey’s post hoc test. *p < 0.05; **p < 0.01; ***p < 0.001; ****p < 0.0001. The data in the dot plots are presented as the mean ± SD. The line on the violin plots depicts the mean

To evaluate antitumor activity, 7aaRGD, 7aaRAE or vehicle (water) were delivered to GL261 tdTomato^+^Luc^+^ gliomas via osmotic pumps for 21 days (Fig. 2A). According to magnetic resonance (MRI) scans at 21 day post-implantation (DPI), tumor growth was not affected by the peptides (Fig. 2B). However, the weights of the 7aaRGD-treated mice significantly increased (Fig. 2C), which is indicative of animal well-being in experimental tumor models. Meanwhile, the weight of the animals that received the vehicle or the control peptide remained unchanged or decreased over time.

To determine the effects of the 7aaRGD peptide on tumor-infiltrating myeloid cells in vivo, we compared the transcriptomic profiles of FACS-sorted glioma-associated CD11b^+^ cells from all experimental groups. At 21 DPI, the percentages of intratumoral CD45^low^CD11b^+^ microglia and CD45^high^CD11b^+^ monocytes/macrophages were similar across treatments (Fig. S4A). However, there were numerous transcriptomic differences reflecting changes in the functionalities of the cells. Among the dowregulated DEGs (differentially expressed genes), some overlapped between the two peptide treatments; however, out of the 563 genes whose expression was upregulated in the 7aaRGD group, only 7 were identified as DEGs in CD11b^+^ cells from 7aaRAE-treated mice, confirming the specificity of the response to the 7aaRGD peptide (Fig. 2D).

Gene Ontology (GO: Biological Process) enrichment analysis (Fig. 2E) revealed that the downregulated genes in the RGD group were related to antigen processing and presentation, negative regulation of lymphocyte activation and negative regulation of immune system processes. Among the upregulated DEGs in the 7aaRGD group, genes involved in the regulation of cell adhesion, T cell activation and leukocyte migration were significantly overrepresented.

The downregulated genes in the 7aaRGD group (Fig. 2F) included *Anpep*, which encodes a membrane-bound enzyme that promotes monocyte adhesion to the endothelium during leukocyte exit into tissues [38], and *Pecam1*, a member of the immunoglobulin superfamily that is involved in leukocyte migration, angiogenesis, and integrin activation. Other dowregulated genes encoded hallmark factors of immunosuppressive macrophages, such as arginase-1 (*Arg1)*, chitinase-like-3 (*Chil3*) and mannose receptor C-type-1 (*Mrc1*). Additionally, decreased levels of *Pdcd1lg2*, *Il4ra* and *Il18bp* were observed. The binding of programmed cell death 1 ligand 2 (PD-L2, encoded by *Pdcd1lg2)* to its receptor PD-1 leads to the inhibition of T cell proliferation and inflammatory cytokine production. *Il4ra* encodes the alpha chain of the receptor for interleukin (IL)-4, which is well known for skewing macrophages toward the alternatively activated phenotype. IL-18-binding protein (encoded by *Il18bp*) is an inhibitor of the proinflammatory cytokine IL18. Moreover, treatment with the 7aaRGD peptide resulted in reduced expression of genes related to antigen processing and presentation, including *H2-Aa* encoding major histocompatibility complex class II (MHC-II) protein and *Ctss* encoding cathepsin S, which participates in the degradation of antigenic proteins to peptides for presentation on MHC-II molecules.

CD11b^+^ cells from the 7aaRGD-treated mice presented increased expression of genes related to T cell activation (Fig. 2G). This group included *CD80*, encoding a co-stimulatory molecule necessary for T cell activation, and *Rsad2*, which is an interferon-stimulated gene involved in IRF7-mediated maturation of mouse dendritic cells (DCs) and their antitumor efficacy. In parallel, genes encoding dedicator of cytokinesis 8 (*Dock8*) and DC-specific C-type lectin receptor DC-SIGN (*CD209a*) were upregulated; these proteins may augment the adaptive immune response by promoting the activation of T cells. Moreover, in CD11b^+^ cells from mice treated with 7aaRGD, genes encoding the following: NEDD4, an E3 ligase involved in the reprogramming of tumor-associated macrophages via CSF1R degradation [39]; CD81, a regulator of the recruitment of NK cells [40]; CD9, a member of the transmembrane 4 superfamily (TM4SF) involved in cell growth, adhesion and motility; the cytokine IL1b and CXCL10, an IFNγ-stimulated chemokine that attracts T cells through binding to its cognate receptor CXCR3, presented increased expression. Genes associated with the cytotoxic activity of T and NK cells, including genes encoding interleukin 2 receptor subunit beta (*IL2rb*), which is involved in T cell immune responses, as well as granzyme A (*Gzma*) and granzyme B (*Gzmb*), which are the major cytolytic factors involved in the antitumor response, were significantly upregulated after 7aaRGD treatment. In addition, 7aaRGD triggered the overexpression of *Fcrls* and *Tmem119*, well-known homeostatic microglia markers, and *Gpr34*, which is upregulated in microglia during inflammation. Another important group of upregulated genes in CD11b^+^ cells from 7aaRGD-treated mice included those encoding several heat shock proteins, which have been implicated in the stimulation of both innate and adaptive immunity [41].

To track cell type alterations induced within brain CD11b^+^ cells by peptide treatments, we deconvoluted bulk RNA-seq gene expression data leveraging CITE-seq transcriptomic signatures of subpopulations of CD11b^+^ cells from naïve and glioma-bearing brains [32] (Fig. S4B-C). Compared with those in the vehicle-treated group, the predicted frequencies of homeostatic microglia (HomMG) increased in response to 7aaRGD treatment, and those of activated microglia decreased, although the latter change was at the boundaries of statistical significance. We hypothetize that the higher proportions of HomMG within GAMs indicated the efficacy of the peptide in mitigating tumor-induced reprogramming. HomMG is the predominant population within CD11b⁺ cells sorted from the healthy (naïve) mouse brain [11, 32], characterized by high expression of canonical microglial genes, such as *Tmem119*, *Fcrls*, and *Cx3cr1* (Fig. S4D), while also maintaining active transcriptional programs. By contrast, tumor-activated microglia exhibit upregulation of genes related to antigen presentation, cytokine production, and phagocytosis/lipid metabolism [32]. Reversing to the homeostatic microglia phenotype could potentially disrupt the cascade of pro-tumorigenic events initiated by microglia activation. In support to our hypothesis, qPCR was performed to validate changes in the canonical microglia markers initially identified by RNAseq, and significantly higher levels of *Fcrls* (and elevated levels of *Cx3cr1* and *Tmem119*) were detected in tumor infiltrating CD11b^+^ cells sorted from the brains of 7aaRGD-treated mice (Fig. S4E). Notably, scRNA-seq feature plots of CD11b⁺-sorted cells from the brains of glioma-bearing animals demonstrate that the expression of target integrin receptor subunits is restricted (αV and β3) or predominant (β5) in microglia. This suggests that microglia serve as the primary target of 7aaRGD-mediated blockade.

To validate the observed transcriptomic changes, suggestive of a switch in the GAMs phenotype upon 7aaRGD administration, we performed immunohistochemical co-staining for IBA1 (ionized calcium-binding adapter molecule-1, a marker of myeloid cells) and Arg1 (arginase-1, a hallmark marker of immunosuppressive/proinvasive GAMs) on brain sections from glioma-bearing mice. The accumulation of Iba1^+^Arg1^+^ positive cells was significantly reduced in the 7aaRGD-treated tumors compared with the control groups (Fig. 2H-I). These data support the findings obtained from the RNA-seq analysis. Although 7aaRGD alone did not reduce the tumor volume or affect the abundance of myeloid cells, it blocked the reprogramming of CD11b^+^ cells into proinvasive, immunosuppressive GAMs. To get further insights, which population of GAMs is mainly responsible for Arg1 production and affected by the treatment, double stainings for Arg1 and Tmem119 (a microglia marker) or galectin-3 (Gal3, a marker for bone marrow-derived infiltrating myeloid cells [32]) were performed. In glioma-bearing brains from the vehicle-treated group, the vast majority of Arg1⁺ cells were Gal3⁺ and localized within the tumor area. Tmem119⁺ cells (microglia) were consistently found at the invasive tumor edge and in the surrounding brain tissue, and displayed low or undetectable levels of Arg1. Tmem119⁺Arg1⁺ cells were rarely observed in the tumor core in either vehicle- or peptide-treated mice. Following 7aaRGD treatment, Gal3⁺ cells exhibited decreased Arg1 expression (Fig. S5A). This suggests that the quantified differences in Arg1⁺Iba1⁺ cells across vehicle-, RAE-, and 7aaRGD-treated groups primarily reflect changes in Arg1 levels within Gal3⁺ myeloid cells (mostly macrophages, as Arg1 is not detected in monocytes and appears in intermediate monocyte-macrophage subpopulation (Fig. S4D and [32]). The higher contribution of monocytes-derived macrophages to elevated Arg1 levels in mouse gliomas, rather than microglia, is consistent with previous findings.

Flow cytometry analysis of the immune infiltrate of gliomas in control and 7aaRGD-treated mice showed increased frequency of CD8 + T cells in the peptide group (Fig. 2J). However, tumor-infiltrating CD8^+^ T cells presented increased expression of the exhaustion marker PD-1 and the infiltration of FoxP3^+^Treg cells into the tumor was unaffected (Fig. 2J and S5B), which could be responsible for lack of antitumor efficacy and provide the rationale for the combination of 7aaRGD with anti-PD-1 immune checkpoint inhibitor.

Another feature strongly associated with the TME is abnormal neovasculature. The structure of blood vessels is disrupted in tumors, leading to the formation of numerous small vessels, which are leaky, collapsed, and disorganized, leading to impaired T cell trafficking, the appearance of hypoxic niches and poor drug delivery [12]. Pathological angiogenesis in GBM is associated with the upregulation of the expression of certain integrins, including αvβ3 and αvβ5. For these reasons, we evaluated the density of the vasculature in GL261 glioma-bearing mice that received 7aaRGD or 7aaRAE peptides intratumorally for 21 days. Lectin staining revealed numerous microvessels in the peritumoral area of vehicle- and 7aaRAE-treated tumor-bearing brains (Fig. 2K-L, Fig. S5C). In the 7aaRGD group, the staining and its quantification demonstrated an increased density of large, elongated vessels in the peritumoral area, consistent with their improved integrity. These findings suggest that 7aaRGD treatment results in vessel normalization. Overall, the phenotype alterations observed in myeloid cells, increased percentage of CD8^+^ T cells and improved vasculature organization indicate immune transition in the TME, which is primed for the complete restoration of antitumor immunity with adjuvant treatment.

### Combining 7aaRGD with PD-1 blockade reduces glioma growth in mice via augmented antitumor immune responses

**Fig. 3:**
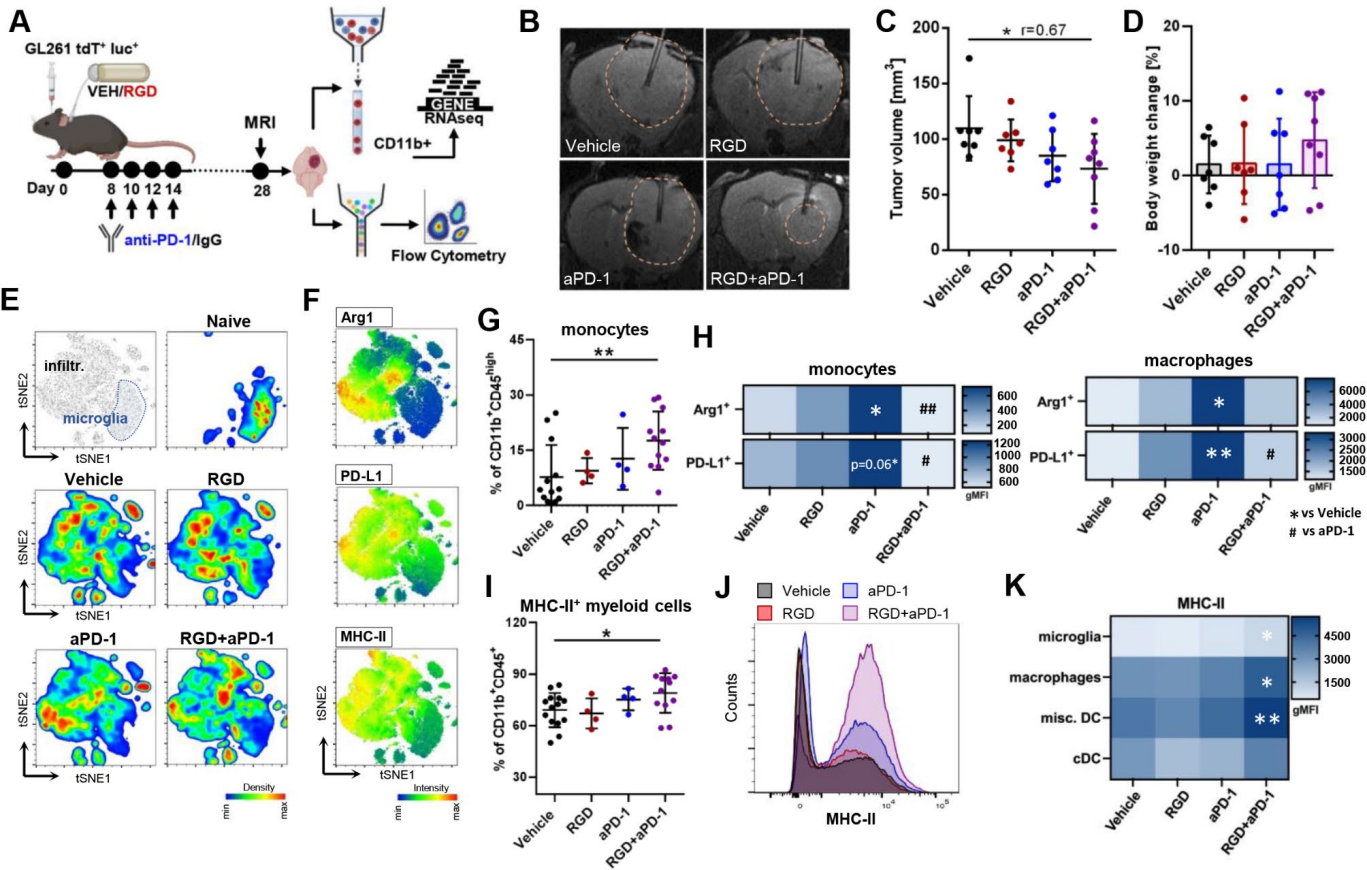
Combining 7aaRGD and anti-PD-1 reduces GL261 glioma growth and instigates changes in the myeloid compartment. (**A**) Scheme of experiments combining 7aaRGD delivery with PD-1 blockade. Mice implanted with GL261 tdTomato + luc + glioma cells received water (vehicle) or the 7aaRGD peptide (RGD) via osmotic pumps for 28 days and anti-PD-1 antibody (aPD-1, 10 mg/kg) or control IgG at day 8, 10, 12 and 14 by i.p. injection. (**B-C**) Tumor volumes were measured by MRI at 28 DPI. The effect size of treatment was assessed with factorial ANOVA; Vehicle-RGD + aPD-1 = 0.67; followed by post-hoc Sidak multiple comparisons test: *p*(Vehicle-RGD + aPD-1) = 0.0128, *n* = 7–8. (**D**) Comparison of body weight between day 0 and 28 DPI; *n* = 7–8; whiskers represent the min–max values, the box extends from the 25th to 75th percentiles, and the line is plotted at the median. (**E**) Unsupervised t-SNE clustering of CD45^+^CD11b^+^ cells from the brains of naïve and Vehicle-, RGD-, aPD-1- and RGD + aPD-1-treated mice. Microglia and infiltrating myeloid cells are separated by manual gating (the first plot from the left). Pseudocolor smooth density plots depict cell clusters of high and low density in red and blue, respectively. (**F**) Heatmap plots showing the levels of functional markers in a blue-to-red scale (low-high). (**G**) Percentages of monocytes (CD45^high^CD11b^+^Ly6C^high^F4/80^low^) in tumor-bearing brains. (**H**) Levels (gMFIs) of Arg1 and PD-L1 in glioma-associated monocytes (CD45^high^CD11b^+^Ly6C^high^F4/80^low^) and macrophages (CD45^high^CD11b^+^Ly6C^low^F4/80^high^); *n* = 4–14. (**I**) Percentages of MHC-II-expressing myeloid cells in tumor-bearing brains and (**J**) a representative histogram. (**K**) Levels (gMFIs) of MHC-II in glioma-associated monocytes (CD45^high^CD11b^+^Ly6C^high^F4/80^low^), macrophages (CD45^high^CD11b^+^Ly6C^low^F4/80^high^), conventional dendritic cells (cDCs; CD45^high^CD11b^−^CD11c^+^) and other DC subsets (misc. DC; CD45^high^CD11b^+^CD11c^+^). Data in all quantitative panels are presented as the mean ± SD. *p < 0.05; **p < 0.01; ***p < 0.001. Statistical analysis was performed using the Mann‒Whitney test or one-way ANOVA, followed by Tukey’s multiple comparison test

The limited efficacy of immune checkpoint inhibitors in GBM is attributed mainly to the highly immunosuppressive TME and altered tumor vasculature impairing T cell trafficking [12]. 7aaRGD treatment blocked the intratumoral reprogramming of myeloid cells into proinvasive and immunosuppressive GAMs and led to blood vessel normalization but also to the increased infiltration of PD-1^+^CD8^+^ T cells. These results prompted us to study whether the 7aaRGD-induced TME and vasculature changes could improve the efficacy of immunotherapy with an anti-PD-1 antibody. The intratumorally delivered 7aaRGD peptide (RGD) was combined with 4 *i.p.* injections of anti-PD-1 (aPD-1 or an isotype control IgG antibody) in immunocompetent mice implanted with GL261 cells (Fig. 3A). MRI revealed significantly reduced tumor volumes at 28 DPI (vehicle vs. RGD + aPD-1: *p* = 0.0128, *r* = 0.67) (Fig. 3B-C). In accordance with the observed reduction in tumor burden, the body weights of 75% of the mice exposed to the combination treatment for 28 days increased over time, whereas in the monotherapy groups, the range of changes in animal weights was similar to that in the vehicle group (Fig. 3D).

Multiparameter flow cytometry analysis was performed on tumor-infiltrating myeloid and lymphoid immune cell populations to dissect changes in the TME composition. At 28 DPI, the total frequency of myeloid cells among all CD45^+^ tumor-infiltrating immune cells as well as the percentages of microglia (CD11b^+^CD45^low^) and infiltrating peripheral myeloid cells (CD11b^+^CD45^high^) did not differ between the vehicle group and the other experimental groups (Fig. S6A-B). However, t-SNE visualization revealed phenotypic shifts in the myeloid compartment upon different treatments (Fig. 3E-F, Fig. S6C). We noticed a significant increase in monocytes (Ly6C^high^F4/80^low^) within the CD11b^+^CD45^high^ population upon RGD + aPD-1 treatment (Fig. 3G). Anti-PD-1 administration led to increased Arg1 and PD-L1 levels in myeloid cells, whereas combination therapy downregulated Arg1 and PD-L1 levels in both monocytes (Ly6C^high^F4/80^low^) and macrophages (Ly6C^low^F4/80^high^) (Fig. 3H). In addition, we observed an increased frequency of MHC-II^+^ cells within the myeloid compartment in the RGD + aPD-1 group compared with the vehicle group (Fig. 3I-J). Upregulated MHC-II levels were detected on microglia, macrophages and subsets of dendritic cells (DCs), mainly CD11b^+^CD11c^+^ DCs but not CD11b^−^CD11c^+^ DCs (Fig. 3K). Augmented MHC-II levels may reflect improved antigen-presenting capacity and facilitate the activation of CD8^+^ T cells.

**Fig. 4:**
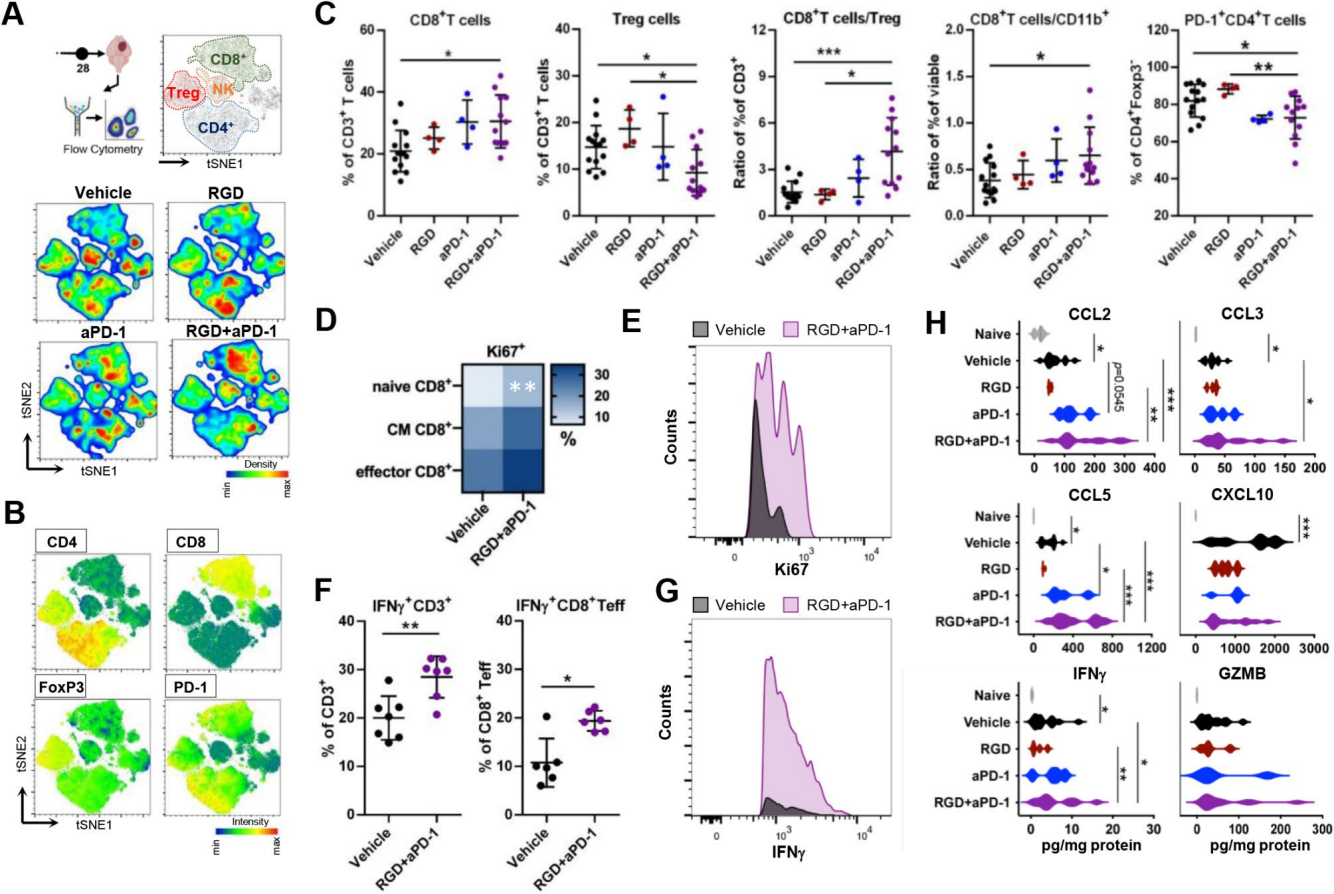
Changes in lymphoid compartments and cytokine levels confer effective antitumor immune responses upon integrin and PD-1 blockade. For the experimental workflow, see Fig. 3. (**A**) Unsupervised t-SNE clustering of CD45^+^CD11b^−^ cells from the brains of Vehicle, RGD-, aPD-1- and RGD + aPD-1-treated mice. The main lymphocyte populations are separated by manual gating (top plot on the right). Pseudocolor smooth density plots depict cell clusters of high and low density in red and blue, respectively. (**B**) Heatmap plots showing the levels of cell identity and functional markers in a blue-to-red scale (low-high). (**C**) Percentages of CD8^+^ (CD8^+^CD4^−^), Tregs (CD4^+^Foxp3^+^CD25^high^), ratio of CD8^+^/Treg cells, percentage of PD-1^+^CD4^+^Foxp3^−^ T cells and ratio of CD8^+^/CD11b^+^cells isolated from tumor-bearing brains in all experimental groups; *n* = 4–14. (**D**) Heatmap showing the percentages of Ki67^+^ cells among effector (CD44^+^CD62L^−^), central memory (CM; CD44^+^CD62L^+^) and naïve (CD44^−^CD62L^+^) CD8^+^ T cells. (**E**) A representative histogram of Ki67^+^ effector CD8^+^ T cells. (**F**) IFNγ production by glioma-infiltrating T cells (CD3^+^) and effector CD8^+^ T cells isolated from the brains of Vehicle- and RGD + aPD-1-treated mice and stimulated with 50 ng/ml PMA + 1 µg/ml ionomycin in the presence of protein transport inhibitors, *n* = 6. (**G**) A representative histogram of IFNγ^+^ effector CD8^+^ T cells. Data in all quantitative panels are presented as the mean ± SD. Statistical analysis was performed using Mann‒Whitney test or one-way ANOVA, followed by Tukey’s multiple comparison test. (**H**) The levels of cytokines were determined in the brain homogenates of naïve and treated mice at 28 DPI using a multiplexed Luminex assay. Violin plots showing the levels of the tested cytokines in pg/mg of total protein. Significance was assessed with One–Way ANOVA and uncorrected Fisher’s LSD multiple comparison test; *N* = 4–12. ***p < 0.001; **p < 0.01; *p < 0.05

Since the detected changes in myeloid cells suggested a proinflammatory switch in the TME of RGD + aPD-1-treated tumors, we investigated how this switch affected the lymphoid compartment. The percentage of CD3^+^ T cells among all CD45^+^ tumor-infiltrating immune cells did not differ between the vehicle group and the other experimental groups (Fig. S6 D). However, t-SNE visualizations of the flow cytometry data revealed quantitative shifts between lymphocyte clusters (Fig. 4A-B, Fig. S6E). The frequency of CD8^+^ T cells was increased in anti-PD-1-treated tumors as compared to untreated tumors and was markedly elevated in the combination group (Fig. 4C and S6F). We noticed a significant decrease in the number of immunosuppressive CD4^+^CD25^high^Foxp3^+^ T regulatory cells (Tregs) in the RGD + aPD-1 group. Consequently, the CD8^+^T cells/Treg ratio, which predicts the response to immunotherapy in cancer patients and experimental mouse models, was increased upon RGD + aPD-1 treatment (Fig. 4C). Moreover, the ratios of CD8^+^ T cells to CD11b^+^ cells were significantly increased in the RGD + aPD-1 group because of the accumulation of cytotoxic T cells, indicating antitumor immune responses in the glioma TME.

We also noted that the percentage of PD-1^+^CD4^+^Foxp3^−^ cells was significantly lower in the RGD + aPD-1-treated mice than in the peptide-alone or vehicle-treated animals (Fig. 4C and S6F). PD-1^+^CD4^+^Foxp3^−^ cells contribute to tumor immune evasion, as they accumulate intratumorally during tumor progression and limit effector CD8^+^ T cell functions. The persistence of elevated frequencies of these cells after PD-1 blockade is a negative prognostic factor in melanoma patients [42].

Tumor-infiltrating T cells usually exhibit a terminally exhausted phenotype, marked by a loss of self-renewal capacity [7]. In the RGD + aPD-1-treated mice, the average frequencies of Ki67^+^ cells among the glioma-infiltrating effector, central memory (CM) and naïve CD8^+^ T cells were increased, indicating augmented proliferation of these cell subsets, with the latter population showing a statistically significant increase compared with the vehicle controls (Fig. 4D-E).

To study whether the effector functions of glioma-infiltrating CD8^+^ T cells are restored upon RGD + aPD-1 treatment, we quantified the production of IFNγ by T cells stimulated ex vivo with PMA and ionomycin. CD3^+^ T cells derived from the RGD + aPD-1 tumors, including effector CD8^+^ T cells, showed increased IFNγ production (Fig. 4F-G). Together, the findings presented here demonstrate profound remodeling of the immune landscape of the glioma TME after RGD + aPD-1 treatment, with a specific shift in the intratumoral reprogramming of infiltrating CD11b^+^CD45^high^ myeloid populations, which results in augmented responses of CD8^+^ T cells.

The reinvigoration of antitumor responses at 28 DPI upon RGD + aPD-1 administration was further evaluated by measuring the levels of pro- and anti-inflammatory cytokines in brain homogenates and sera (Fig. 4H, Fig. S6G-H). C-C motif chemokine ligand (CCL) 2, CCL3 and CCL5 levels were increased in brain homogenates from aPD-1-treated mice (vs. the vehicle group), and even higher levels were detected in RGD + aPD-1-treated animals. CXCL10 levels were elevated (compared with those in naïve mice) in the brains of glioma-bearing mice independent of treatment. IFNɣ, granzyme B and TNFα, which are produced by activated immune cells and linked to antitumor immune responses, were elevated in the aPD-1 and RGD + aPD-1 groups as compared to the vehicle group, with the levels of IFNɣ, the master immune effector cytokine, being significantly higher in the RGD + aPD-1 group. Interestingly, the levels of these cytokines measured in the sera of the same animals remained unchanged upon treatments, except for decreased levels of CCL2 in the RGD + aPD-1 group (Fig. S6H). CCL2 and CCL5 are involved in monocyte attraction to tumors [43], and CCL5 is crucial for DCs recruitment and, in conjunction with CXCL9/10, generates the main chemotactic ques for effector T cells [44, 45]. CCL3 is considered a marker of proinflammatory macrophages and leads to improved DCs and T cell responses in the TME [46]. The observed contextual changes in cytokine levels support the notion that the proinflammatory TME in experimental gliomas is restored upon RGD + aPD-1 treatment.

Antitumor immune responses in aPD-1- and RGD + aPD-1-treated gliomas evolved with time, as at an earlier time point 21 DPI, the tumor volume did not change upon treatment (Fig. S7A-B), and the myeloid compartment was dominated by Arg1^+^ and PD-L1^+^ immunosuppressive macrophages (Fig. S7C-D). Interestingly, the observed effects were specific to the tumor niche, as peripheral monocytes and granulocytes isolated from the spleen were not affected (Fig. S7E-F). At 21 DPI, there were already some qualitative changes in the lymphoid compartment of the TME (Fig. S7G-J). The intratumoral levels of effector CD8^+^ T cells were significantly increased after aPD-1 and RGD + aPD-1 treatments. Concurrently, the percentage of Tregs remained unchanged in the tumor but was elevated at the periphery in the aPD-1 and RGD + aPD-1 groups, counteracting the revival of antitumor responses. Similar evolution of responses to ICIs with initial upregulation of immunosuppression as a feedback loop has been previously reported [35, 47].

### GAMs from 7aaRGD + anti-PD-1-treated mice are transcriptionally reprogrammed for antitumor responses

**Fig. 5:**
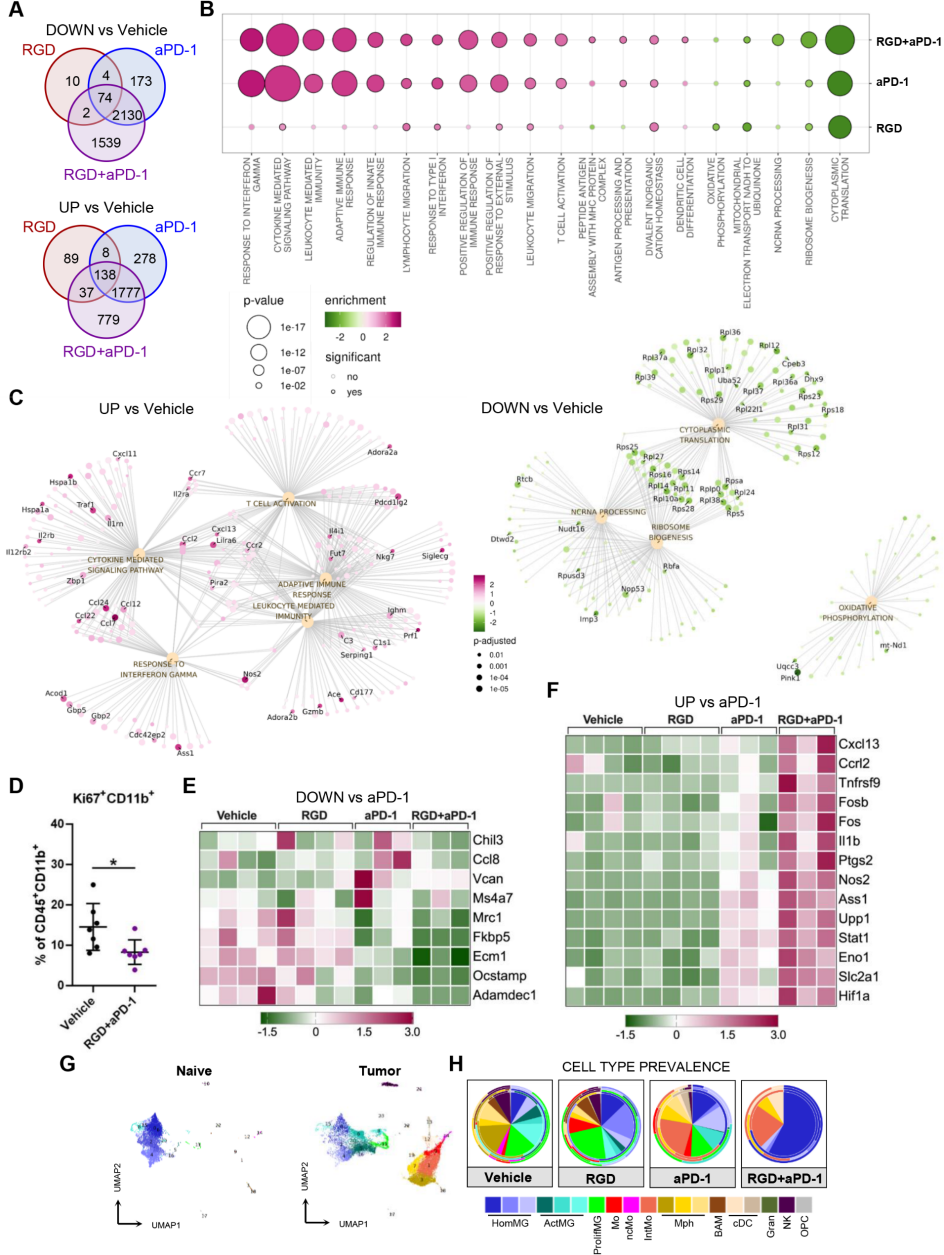
The transcriptomic profiles of CD11b^+^ cells from 7aaRGD + anti-PD-1-treated mice reveal reduced expression of tumor-supportive genes and activation of antitumor response. Gene expression profiling using RNA-seq of CD11b^+^ cells from the tumor-bearing hemispheres of mice from all experimental groups at 28 DPI. (**A**) Venn diagrams showing a number of down- and upregulated DEGs in the 7aaRGD (RGD), anti-PD-1 (aPD-1) and RGD + aPD-1 groups compared with the Vehicle group. (**B**) Functional enrichment of GO biological processes for up- and downregulated genes in the RGD + aPD-1 group compared with the Vehicle group. (**C**) Graphical representation of selected overrepresented GO terms among DEGs in the RGD + aPD-1 group versus the Vehicle group. The dots represent genes, the lines represent membership in the given term; the top 40 genes with outstanding fold changes and *p* values are labeled. (**D**) Percentages of proliferating (Ki67^+^) myeloid cells isolated from the brains of Vehicle- and RGD + aPD-1-treated mice. Statistical analysis using the Mann‒Whitney test; *p < 0.05. (**E, F**) Z-score heatmaps representing the relative gene expression in CD11b^+^ cells isolated from the brains of glioma-bearing mice. The selected down- (**E**) and upregulated genes (**F**) comprise the top DEGs between RGD + aPD-1 and aPD-1. (**G-H**) Cell type prevalence inferred from deconvolution of bulk RNA-seq data of CD11b^+^ cells sorted at 28 DPI. Deconvolution was based on transcriptomic signatures (see Fig. S4) of myeloid cell clusters from naïve and glioma-bearing brains (**G**). The predicted cell type abundance is presented on pie charts (**H**), with average values in the center and the cell prevalence in each repetition indicated on the external rims

In order to deeply characterize the responses of GAMs to integrin and/or immune-check-point blockade, transcriptome profiling of CD11b^+^ cells isolated from the brains of tumor-bearing mice at 28 DPI was performed using RNA-seq. The most profound changes versus the vehicle group were detected upon the combination treatment with 7aaRGD and anti-PD-1 (RGD + aPD-1) (Fig. 5A). The GO analysis of the DEGs in this group revealed the upregulation of several pathways related to inflammatory and antitumor responses, such as T cell activation, cytokine-mediated signaling pathway, adaptive immune response, response to interferon gamma, leukocyte migration and antigen processing and presentation (Fig. 5B). These categories included genes coding for co-stimulatory molecules and markers delineating mature APC functions (*Icam1*,* CD40*,* and CD86*), chemokines, cytokines and their receptors (*Ccl7*,* Ccl5*,* Ccl2*,* Cxcl9*,* Cxcl10*,* Cxcl13*,* Il2ra*,* Il2rb*,* and Ccr7*) and tumoricidal factor machinery (*Nos2*,* Ass1*,* Prf1*,* and Gzmb*) (Fig. 5C). The downregulated genes were associated with GO terms related mainly to translation and cell division (Fig. 5B), which was in line with the significant decrease in Ki67^+^CD11b^+^ proliferating GAMs upon RGD + aPD-1 treatment (Fig. 5D). The downregulated genes included *Gpnmb*, encoding transmembrane glycoprotein nonmetastatic B (GPNMB), which is overexpressed in GAMs in humans and mice and is associated with poor patient prognosis [28, 48]. GPNMB produced by macrophages plays a crucial role in proneural‒mesenchymal transition through immune cell‒tumor interplay, and GPNMB-high macrophages impair T cell activation by DCs [49]. Additionally, genes involved in mitochondrial electron transport and oxidative phosphorylation were downregulated, indicating a metabolic switch toward proinflammatory myeloid cells (Fig. 5B-C). These data are consistent with the reduced protumoral activation of GAMs and their transition to the proinflammatory state.

The transcriptomic responses in tumor-infiltrating CD11b^+^ cells from the aPD-1 alone and aPD-1 + RGD groups overlapped to a great extent. However, the downregulated genes in the RGD + aPD-1 group compared with the aPD-1 group included known markers of a proinvasive phenotype and suppressors of a proinflammatory switch in macrophages, such as *Chil3*, *Mrc1*, *Fkbp5*, *Ms4a7*, *Vcan*, *Ecm1*, *Ccl8* and *Ocstamp* (Fig. 5E). Among the genes significantly upregulated in RGD + aPD-1 vs. aPD-1, the following genes were identified: *Ptgs2*,* Nos2*,* Ass1*,* Il1b*, *Stat1* (encoding mediators and enzymes vital for the macrophage proinflammatory immune response [50]), *Cxcl13* (encoding a B-cell chemoattractant), *Ccrl2* (a predictive indicator of robust antitumor T-cell responses, selectively expressed in TAMs with an immunostimulatory phenotype in humans and mice [51]), *Slc2a1* and *Upp1* (related to glycolysis) and *Tnfrsf9* (encoding CD137/4-1BB), whose expression on human monocytes/macrophages leads to their metabolic and functional reprogramming and enhances their tumoricidal activity [52] (Fig. 5F). These transcriptomic changes in glioma-associated CD11b^+^ cells highlight the superior efficacy of RGD + aPD-1 treatment over aPD-1 monotherapy.

Finally, through bulk RNA-seq deconvolution (leveraging the abovementioned CITEseq data [32]), we inferred the cell type abundance within the immunosorted glioma-associated CD11b^+^ cells from the animals in each group (Fig. 5G-H, Fig. S8). We detected a significantly higher prevalence of homeostatic microglia and a reduced abundance of activated microglia within CD11b^+^ cells in the RGD + aPD-1-treated mice as compared to the vehicle group. Intermediate monocyte and DC signatures were enriched in the aPD-1 and RGD + aPD-1 groups, whereas decreased macrophage scores characterized the response to RGD and RGD + aPD-1. CD11b^+^ cells from the RGD + aPD-1 group presented a predominance of homeostatic microglial signatures, indicative of the phenotype observed in the healthy mouse brain, which corroborated the lower tumor burden in these mice.

### Targeting intergin signalling has translational value

**Fig. 6:**
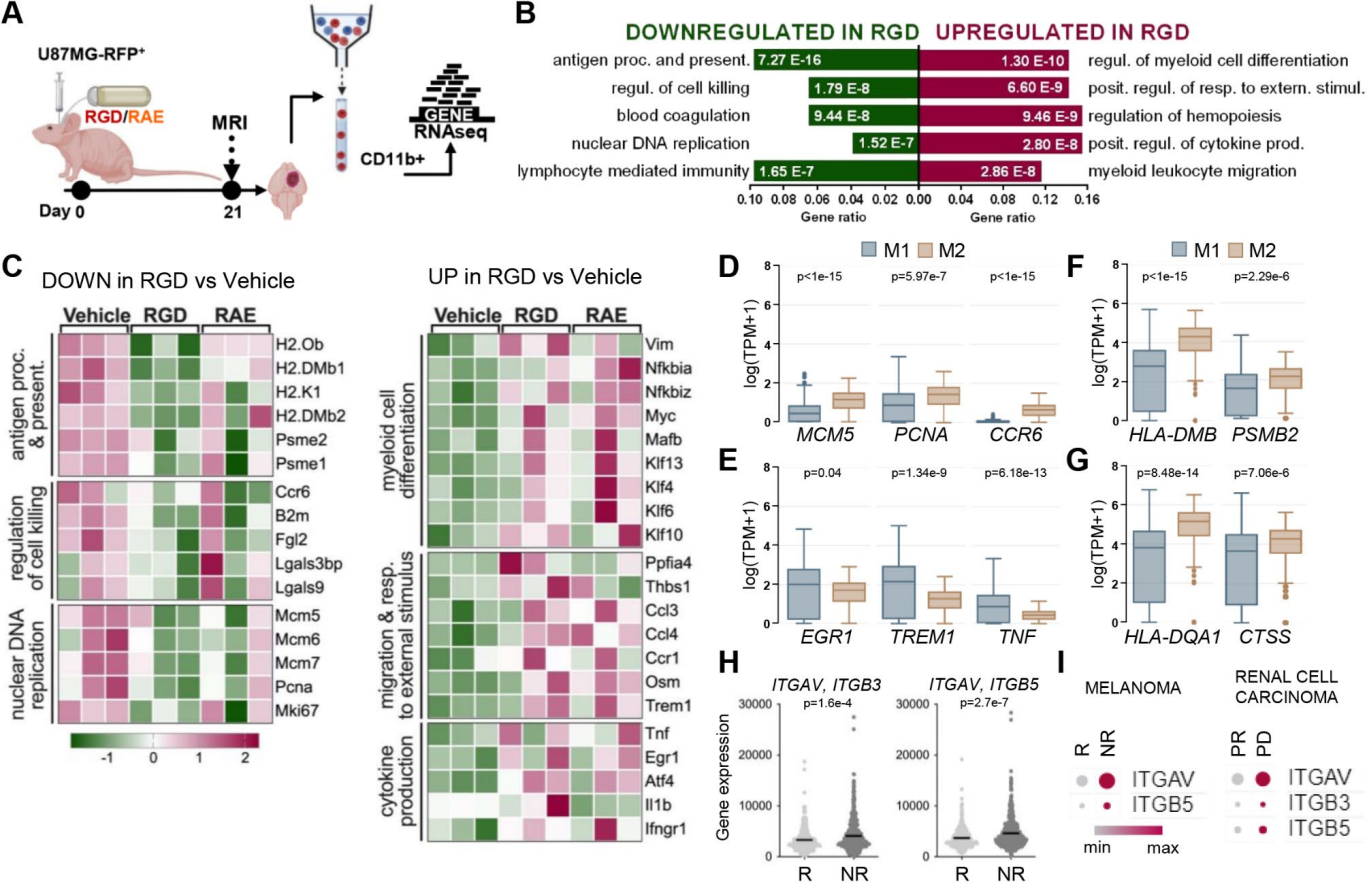
Integrin blockade counteracts the reprogramming of GAMs by human gliomas in mice and emerges as a strategy for improving the ICI immunotherapy response. (**A**) Scheme of the experimental pipeline. Athymic nude-Foxn1nu mice were orthotopically implanted with U87-MG-RFP^+^ human glioma cells and received water (Vehicle), 7aaRGD (RGD) or control peptide 7aaRAE (RAE) via osmotic pumps. (**B**) Functional enrichment analysis with GO biological process terms for down- and upregulated genes in CD11b^+^ cells isolated at 21 DPI from glioma-bearing brain hemispheres of mice treated with 7aaRGD as compared to Vehicle. The enriched GO pathways are shown; the size of the bars indicates gene ratios (the number of genes annotated to the pathway/total number of DEGs). (**C**) Z-score heatmaps with selected up- and downregulated genes in CD11b^+^ cells from 7aaRGD- vs. Vehicle-treated animals. (**D-G**) Differential gene expression in classically (M1) and alternatively (M2) activated macrophages inferred from bulk RNA-seq data from GBM samples (TCGA) using the quanTIseq deconvolution tool (via Gepia2021). Human analogs of the selected genes identified as downregulated (**D, F, G**) or upregulated (**E**) in GAMs after 7aaRGD treatment in nude (**D, E, F**) and C57BL/6 (**G**) mice. (**H**) Levels of *ITGAV/ITGB3* and *ITGAV/ITGB5* in pretreatment tumor biopsies from ICI therapy responders (R) and non-responders (NR) (according to ROCplotter). Statistical analysis was performed using the Mann‒Whitney test. (**I**) *ITGAV*, *ITGB3*, and *ITGB5* expression in tumor-associated myeloid cells from ICI-treated patients (R, responders; NR, nonresponders; PR, partial response; PD, progressive disease). Generated via Single Cell Portal

The responses of brain myeloid cells to patient-derived orthotopic xenografts in immunodeficient mice follow similar patterns as those in syngeneic experimental glioma models [53]. The 7aaRGD peptide effectively blocked the invasion of human glioma cells in mouse and human microglia co-cultures (Fig. 1). Thus, we tested whether intratumorally administered 7aaRGD can modify the myeloid TME in intracranial human gliomas in immunodeficient mice. U87-MG RFP^+^ tumor-bearing mice received vehicle, 7aaRGD or the control peptide 7aaRAE via osmotic micropumps (Fig. 6A). 7aaRGD did not reduce tumor growth compared with the other treatments (Fig. S9A), and the changes in animal body weight over the course of the experiment were within the same range in all the groups (Fig. S9B). Additionally, the percentages of microglia and macrophages in the tumor-bearing hemispheres of the mice at 21 DPI were similar across all the experimental groups (Fig. S9C). However, gene expression profiling of FACS-sorted CD11b^+^ cells revealed changes in myeloid cells phenotypes upon 7aaRGD treatment (Fig. 6B-C).

GO functional enrichment analysis of the DEGs revealed that genes involved in antigen processing and presentation, regulation of cell killing, blood coagulation and DNA replication were downregulated in the 7aaRGD group compared with the vehicle group (Fig. 6B). Among these genes, we identified minichromosome maintenance protein 5 (*Mcm5*) and its analogs (*Mcm6*, *Mcm7*), proliferating cell nuclear antigen (*Pcna*) and *Ki67*, which indicates decreased proliferation of GAMs (Fig. 6C). Genes related to antigen processing and presentation, encoding the components of MHC class I (*B2m* and *H2-K1*) and MHC class II (*H2-Ob*,* H2-DMb1*,* H2-DMb2)*, as well as genes implicated in the regulation of cell killing, such as chemokine receptor 6 (*Ccr6*) expressed on immature dendritic cells, galectin-3 binding protein - a negative regulator of NF-κB activation (*Lgals3bp)*, and galectin-9 (*Lgals9)*, which contributes to M2-type macrophage polarization, were downregulated in the 7aaRGD group (Fig. 6C).

The upregulated genes in glioma-associated CD11b^+^ cells from the 7aaRGD-treated mice were related to myeloid cell differentiation, response to external stimulus, myeloid leukocyte migration and cytokine production (Fig. 6B). We detected high expression of *Il1b* and *Tnf* cytokine-encoding genes and *Ifngr1*, which encodes the ligand-binding chain of the interferon-gamma receptor. The upregulated genes related to myeloid differentiation, activation and polarization included *Vim* (induced in TNFα-activated macrophages), *Myc*, *Mafb*, *Nfkbia*, and *Nfkbiz*, as well as genes encoding several Kruppel-like factors (*Klf4*, *Klf6*, *Klf10*, and *Klf13*) [54], indicating reprogramming of CD11b^+^ cells in 7aaRGD-treated mice. Interestingly, 7aaRGD treatment induced the expression of genes involved in the response to external stimulus, including oncostatin M (*Osm*), which is released by proinflammatory macrophages and has antitumor potential [55], and genes such as *Ccl3*,* Ccl4* and *Ccr1*, which are critical for the recruitment of leukocytes to the site of inflammation, and *Trem1*, encoding triggering receptor expressed on myeloid cells-1 (TREM1), a proinflammatory receptor that amplifies antitumor immune responses [56] (Fig. 6C).

In GBM samples from the TCGA database, human analogs of the genes downregulated in CD11b^+^ in response to 7aaRGD in U87-MG-RFP^+^ gliomas (including genes involved in antigen presentation in both experimental models) were inferred to be expressed in alternatively (M2) activated macrophages (Fig. 6D, F-G), and those with upregulated expression in the 7aaRGD group were predominantly associated with classically (M1) activated macrophages (Fig. 6E), as predicted using quanTIseq deconvolution of bulk RNA-seq data via GEPIA2021. These data indicate the ability of 7aaRGD to block protumoral myeloid cell reprogramming induced by human glioma cells and to induce the re-emergence of macrophages with an antitumor phenotype.

According to analysis leveraging ROC Plotter (https://www.rocplot.com/immune [57]),, the *ITGAV*/*ITGB3* and *ITGAV*/*ITGB5* signatures in pretreatment biopsies were significantly higher in non-responders than in responders to ICI immunotherapy in various types of cancers (Fig. 6H). Congruently, exploration of scRNA-seq data (via Single Cell Portal) revealed that elevated levels of *ITGAV*,* ITGB3 and ITGB5* in myeloid cells are negative predictors of ICI therapy response in melanoma and renal cell carcinoma patients (Fig. 6I). These findings suggest that increased expression of these RGD-motif binding integrins confers resistance to immunotherapies.

## Discussion

Patients with GBM rarely respond to ICIs, with clinical benefits reported in less than 10% of patients and only in a neoadjuvant setting [15, 58, 59]. In this study, we propose a new approach for anti-glioma therapy based on combining integrin blockade with PD-1 blockade. We provide strong evidence that the designed synthetic 7aaRGD peptide, which targets SPP1/integrin-mediated signaling and has been previously validated in in vitro experiments [21], blocks tumor-induced reprogramming of myeloid cells in vivo. The intratumoral administration of 7aaRGD results in the conversion of a “cold” to a “hot” TME and the stabilization of neoangiogenesis, boosts the innate and adaptive immune responses elicited by anti-PD-1 antibodies and leads to augmented antitumor activity.

GAMs are the major immune component of GBM, and they contribute to tumor progression and impair antitumor responses [6, 7, 11]. Preclinical data from rodent glioma models, including ours, highlight the importance of αvβ3/αvβ5 integrin signaling in recruitment [23] and phenotype decision-making in GAMs [21, 22]. Herein, we provide compelling evidence that 7aaRGD, the integrin-blocking peptide, hinders glioma–microglia interactions, inhibits the microglia-dependent invasion of human and mouse glioma cells in vitro and prevents the protumorigenic reprogramming of GAMs in vivo. Through transcriptome profiling, we demonstrated that integrin blockade using 7aaRGD induced a phenotypic switch in GAMs in GL261/C57BL5 and U87-MG-RFP/Nude-Foxn1nu mouse models, corroborating our previous data showing the antitumor effects of SPP1 or MFG-E8 knockdown in rats [21]. Hallmark protumorigenic genes (including *Arg1*, *Mrc1*, and *Il18bp*) were downregulated, and genes linked to T cell chemotaxis, cytotoxic NK function and proinflammatory cytokine production were upregulated in GAMs from 7aaRGD-treated mice. In line with the transcriptomic data, GL261 tumors in 7aaRGD-treated mice were significantly less infiltrated by Arg1^+^ myeloid cells as compared to controls. Arginase deprives arginine, which is auxotrophic for T cells. A profound decrease in the frequency of Arg1^+^ GAMs mitigates one of the major obstacles to T cell survival in the TME. In our recent study, arginase inhibition combined with an anti-PD-1 antibody effectively reduced glioma growth in mice [35], prompting us to hypothesize that 7aaRGD-driven phenotype switch of GAMs may also contribute to an improved response to ICIs and potentially to other immunotherapies. Notably, 7aaRGD-treated mice exhibited an increased frequency of CD8 + T cells. However, the elevated expression of the exhaustion marker PD-1 on these cells, along with a high abundance of Treg cells and other tumor-promoting or immunosuppressive mechanisms, may explain why 7aaRGD alone failed to reduce tumor growth despite its effects on the immune tumor microenvironment. Targeting myeloid cells by disrupting the CCL2/CCR2 axis, which regulates monocyte and macrophage recruitment to the tumor microenvironment, can limit the infiltration of pro-tumorigenic myeloid cells and enhance anti-tumor immune responses in experimental gliomas. However, CCR2 deficiency alone was insufficient for significant tumor regression but proved more effective when combined with checkpoint inhibitors [16].

Owing to the intricate relationship between the innate and adaptive immune systems, the response of GAMs to 7aaRGD treatment was more pronounced in immunocompetent mice than in mice devoid of the adaptive immune system. Interestingly, the top common downregulated pathways in the GAMs sorted from the 7aaRGD-treated mice in both models were related to antigen processing and presentation. Although this function is a prerequisite for the antitumor immune response, in the absence or malfunction of co-stimulatory molecules (such as B7-H2, B7-1, B7-2), antigen presentation can render the recipient T cells anergic [60, 61]. Downregulation of B7 molecules is frequent in tumors and disables a proper T cells activation. Glioma-infiltrating microglia/macrophages from postoperative tissue samples of glioma patients expressed surface MHC-II but lacked the expression of the co-stimulatory molecules CD86, CD80, and CD40, which are critical for T cell activation [62]. MHC-II-expressing blood-derived myeloid cells in GL261 tumors in mice displayed a bona fide immunosuppressive and reduced co-stimulatory phenotype [63]. Transcriptomics profiling of myeloid cells in GBM showed downregulation of antigen processing and presentation genes and it was interpreted that myeloid cells in GBM acquire the M0 state [64]. Whether the 7aaRGD-dependent decrease in antigen presentation in the immunosuppressed TME may play a role in the subsequent response to immunotherapy requires further studies. However, we cannot exclude the possibility that the downregulation of genes related to antigen processing and presentation pathways in sorted CD11b⁺ cells from 7aaRGD-treated tumors (bulk RNA-seq data) is due to a higher proportion of homeostatic microglia rather than tumor-activated microglia, which exhibit elevated expression of antigen presentation genes (Fig. S4B-C, E).

The αvβ3 and αvβ5 integrin receptors are strongly overexpressed on proangiogenic endothelial cells, including those that form tumor-associated blood vessels in GBM, and inhibitors targeting these integrins, such as the cyclic peptide cilengitide (CIL), act as anti-angiogenic agents [25]. We report that the intratumoral delivery of 7aaRGD resulted in improved vessel density. This finding corroborates the paradoxical proangiogenic effect reported with CIL (at low doses), which led to vascular normalization, i.e., remodeling of the structurally and functionally immature tumor vasculature, resulting in improved blood flow and drug delivery [65]. This phenomenon might contribute to improved antitumor effects of radiotherapy or chemotherapy upon co-administration with CIL [26, 66], and the clinical benefit apparently observed in some GBM patients with MGMT promoter methylation in a phase III clinical trial, in which CIL was added to standard chemoradiotherapy [67]. Vascular normalization transiently induced by anti-angiogenic therapy promotes the infiltration of T lymphocytes, diminishes hypoxia and thereby immunosuppression within tumors [68–70]. Anti-VEGF drugs enhance the efficacy of ICIs in preclinical settings, including in GBM models [12, 13]. Hence, the increased density of structurally improved blood vessels observed upon intratumoral delivery of 7aaRGD might have contributed to the increased response to anti-PD-1 immunotherapy in glioma-bearing mice.

Profiling the immune landscapes of human and mouse tumors has revealed that the scarcity of activated cytotoxic T cells, the plenitude of suppressive myeloid cell subsets and poor antigen presentation in the brain are the major obstacles to effective immunotherapy in GBM [17, 47]. According to a recent single-cell study, neoadjuvant PD-1 blockade induced T cell and cDC1 activation but failed to overcome the immunosuppressive GAMs in recurrent human GBM [71]. Therefore, several combination therapies involving the blockade of infiltration or the re-education of suppressive myeloid cells with ICIs have been tested in preclinical settings [16, 19, 47, 72]. In the present study, we demonstrated the synergistic effects of intratumorally delivered 7aaRGD and systemic administration of anti-PD-1 antibodies in mice harboring syngeneic GL261 tumors. Anti-PD-1 treatment, either alone or in combination, led to a decrease in the expression of protumoral marker genes (such as *Gpnmb* and *Mrc1*) and upregulated the expression of genes related to antigen presentation, interferon signaling, the innate immune response and the secretion of T cell chemotactic factors. However, ICI monotherapy induced the previously reported negative feedback response [73] characterized by the upregulation of Arg1 and PD-L1 on myeloid cells and sustained levels of Treg cells, counteracting the reactivation of T cells after immunotherapy. PD-L1 expression on macrophages has been associated with poor survival and resistance to immunotherapy in GBM patients [47, 74]. Disruption of the feedback response and reduced expression of immunosuppressive molecules in GAMs upon co-administration of 7aaRGD likely contributed to the efficacy of the combined treatment. Accordingly, the transcriptomes of glioma-associated CD11b^+^ cells from aPD-1- and RGD + aPD-1-treated mice revealed a complete phenotypic switch only in the latter group. Intratumoral microglia and, more importantly, blood-derived macrophages/DCs from RGD + aPD-1 mice presented elevated levels of MHC-II. MHC-II-restricted antigen presentation by cells of the myeloid compartment, particularly those infiltrating from the periphery but not microglia alone, is required for the response to ICIs and correlates with favorable clinical outcomes in GBM patients receiving anti-PD-1 therapy [63].

Reprogramming of GAMs in RGD + aPD-1-treated mice was associated with improved T-cell immune surveillance within the TME, reflected by increased frequency and proliferation of intratumoral CD8^+^ T cells, reduced numbers of Tregs and increased ratios of CD8^+^/Treg and CD8^+^/CD11b^+^ cells. Moreover, RGD + aPD-1 treatment promoted IFNγ production by tumor-infiltrating T cells, including CD8^+^ effector T cells, and elevated IFNγ intratumoral levels. Consistent with the augmented antitumor response, the highest concentrations of cytolytic granzyme B and TNFα were detected in the immunostimulatory milieu of RGD + aPD-1-treated tumors, which can attract additional T cells and professional antigen-presenting cells (e.g., dendritic cells) via elevated CCL3, CCL5 and CXCL10. Thus, the combined treatment reshaped the glioma immune landscape and incapacitated several mechanisms that dampen the proper action of T cells in the TME, resulting in the increased T cell proliferation and activation needed for functional antitumor responses.

GBM remains an incurable disease, and improving its treatment remains a paramount challenge for clinicians and researchers. Here, we show the synergy between two therapeutic modalities that have failed individually: an immune checkpoint and integrin blockade. We revealed that the 7aaRGD peptide modifies the function of GAMs and the tumor neovasculature. The bimodal action of the peptide reshapes the glioma TME and facilitates the successful revival of the antitumor host response with ICIs. While the results show new therapeutic opportunities for GBM patients, some limitations to our work should be considered. In all experiments, microglia vs. bone-marrow/blood-derived myeloid cells were distinguished using CD11b and CD45 level alone, and CD11b^+^CD45^high^ cells, considered as infiltrating myeloid cells, were assumed to be mainly monocytes/macrophages, while CD11b^+^CD45^high^ population contains other myeloid cells: MDSCs, dendritic cells and neutrophils. Our previous work [11, 32] confirmed that monocytes and macrophages are by far the dominant population within these cells in the GL261 glioma model. However, a lack of additional markers defining DC population (such as Flt3, CD103, CD209a, Xcr1) can be considered as a limitation of this study. The synergistic effects of ICIs and 7aaRGD could not be tested in immunodeficient mice; however, we demonstrated the efficacy of 7aaRGD in blocking the reprogramming of myeloid cells in human U87-MG gliomas in vivo and in co-culture models. 7aaRGD was delivered throughout the experiment to precondition the immune-suppressed TME via integrin antagonism. Investigations into the optimal duration of treatment and the best timing of administration of PD-1 inhibitors may further enhance the efficacy of the combinatorial approach. A potential caveat of our strategy is the limited peptide stability upon systemic administration. The development of modified peptides or intratumoral delivery systems could overcome such challenges in the future.

GBMs are notoriously recurring tumors. Due to their diffusive character their total resection is practically impossible. We consider the presented scheme of 7aaRGD administration a model of post-operative therapy aiming to prevent recurrence of the tumor. Intratumoral delivery along with convention enhanced delivery has been tested in completed (NCT00038441, NCT01491893, NCT02798406) and active (NCT05324501, NCT02208362, NCT03389230) clinical trials, especially in a postoperative, anti-recurrence scenario. The limitations of intratumoral administration, which is an invasive procedure itself, are numerous. We and others are working of alternative pathways of the peptide delivery i.e. nose-to-brain administration of a “naked” peptide or in a nanovector [75], which can be a more flexible tool to treat GBM in a therapeutic setting.

Integrin-coding genes are evidently upregulated in GBM and expressed on myeloid cells, pericytes and endothelial cells, and their high expression is associated with worse outcomes for patients (Fig. 1B, C) and a greater risk of ICI therapy failure (Fig. 6H, I). Therefore, αvβ3/5 integrins are valid therapeutic targets in GBM. The successful use of integrin-blocking agents requires proper patient stratification on the basis of the ligand (e.g., SPP1) or target integrin expression. In a retrospective study, higher αvβ3 protein levels were associated with improved survival in GBM patients treated with CIL [27]. The presented results encourage further studies of integrin signaling inhibition in human GBM and indicate its potential clinical application. Finally, reinvestigating patient responses to previous anti-integrin therapies from the perspective of their impact on myeloid cells may shed light on potential benefits from the combination of integrin blockers with immunotherapy.

## Conclusions

We demonstrate that a designer 7aaRGD peptide that blocks SPP1/integrin signaling prevents the emergence of immunosuppressive GAMs, and normalizes the tumor vasculature in experimental gliomas. Due to its bimodal action, the peptide shifts the glioma TME from a “cold” toward a “hot” phenotype. Combining integrin blockade with ICIs reinvigorates antitumor immunity by boosting both innate and adaptive immune responses and leads to eradication of the tumors. These results provide a new perspective for the treatment of GBM patients and pave the way for improving immunotherapy outcomes in GBM and other cancers.

## Electronic supplementary material

Below is the link to the electronic supplementary material.

Additional file 1: Fig. S1 shows gene expression data of RGD motif-binding integrins in human glioma samples from public repositories and its correlation with patient survival. Fig. S2 presents the effect of the 7aaRGD peptide on gene expression in microglia-glioma co-cultures, shows representative pictures from Matrigel assay with two additional cell lines and presents the effect of the 7aaRGD peptide on the viability and proliferation of glioma and microglia cells. Fig. S3 describes stability studies and biodistribution of the 7aaRGD peptide delivered in osmotic pumps. Fig. S4 presents the profiles of myeloid cell in the brain of 7aaRGD-treated GL261 glioma-bearing mice analysed by flow cytometry and inferred from the deconvolution of bulk RNA-seq data of sorted tumor-infiltrating CD11b^+^ cells. Fig. S5 presents representative images from Gal3/ Tmem119 and Arg1 doublestaings. Fig. S6 shows extended data on characterization of the immune microenvironment of GL261 gliomas upon 7aaRGD or/and anti-PD-1 treatment. Fig. S7 presents the data on immune cells phenotyping in the TME and spleens at 21 DPI in glioma-bearing mice. Fig. S8 presents the profiles of intratumoral myeloid cells in different treatment groups inferred from the deconvolution of bulk RNA-seq data. Fig. S9 shows key parameters of the response to 7aaRGD-treatment in U87-MG-RFP + glioma-bearing mice. Fig. S10 and Fig. S11 present the gating strategy used for FACS-sorting and flow cytometry analysis, respectively. Table S1 lists all antibodies used for immunohistochemistry and flow cytometry stainings

## Data Availability

The RNA-seq datasets supporting the conclusions of this article have been deposited in the NCBI Gene Expression Omnibus with the GSE266110 identifier. The materials generated in this study can be requested from BK or AEM.

## Abbreviations

GBM: Glioblastoma. IDH1/2wt diffuse glioma
GAMs: Glioma–associated microglia and macrophages
ICIs: Immune checkpoint inhibitors
TME: Tumor microenvironment
